# Co(II), Cu(II), and Ni(II) Coordination Complexes: Synthesis, Characterization, Experimental, and Computational Study on Potential Antiplasmodial Activity

**DOI:** 10.1002/cmdc.70347

**Published:** 2026-06-22

**Authors:** David Ezenarro‐Salcedo, Daniela Fonseca‐López, Alexander Patiño‐Cubides, María C. Velasco‐Pareja, María F. Yasnot‐Acosta, Camilo Serrano‐Sterling, Ana Rodríguez, Mario A. Macías, Adrian L. Orjuela, Augusto Valderrama‐Aguirre, John J. Hurtado

**Affiliations:** ^1^ Grupo de investigación en Química Inorgánica, Catálisis y Bioinorgánica (GUIQUICB) Departamento de Química Universidad de los Andes Bogotá Colombia; ^2^ Grupo de Investigaciones Microbiológicas y Biomédicas de Córdoba (GIMBIC) Laboratorio de Salud Pública Universidad de Córdoba Montería Colombia; ^3^ Doctorado en Medicina Tropical Universidad de Cartagena Cartagena Colombia; ^4^ Cristalografía y Química de Materiales (CrisQuimMat) Departamento de Química Universidad de los Andes Bogotá Colombia; ^5^ Department of Microbiology New York University School of Medicine New York City New York USA; ^6^ Center for Biodiversity and Drug Discovery Instituto de Investigaciones Científicas y Servicios de Alta Tecnología (INDICASAT AIP) Panamá República de Panamá; ^7^ Director of the Biomedical Research Institute Group Departamento de Ciencias Biológicas Universidad de los Andes Bogotá Colombia

**Keywords:** antiplasmodial activity, coordination complexes, crystallization, imidazole‐derived ligand, X‐ray diffraction

## Abstract

In this work, the synthesis and structural characterization of coordination complexes **C1** (Co−Cl), **C2** (Cu−Cl), **C3** (Co−Br), and **C4** (Ni−Cl) with the ligand 2‐(*tert*‐butoxy)‐6‐(1H‐imidazol‐1‐yl)pyridine (**L**) was carried out. The X‐ray diffraction reveals the presence of four equatorial L and two axial halogens as coligands. The synthesized complexes crystallize in a *P*−1 space group. Topological analysis using ToposPro assigned the discrete 1,6M7‐1 topology, in which ZA1 and ZA2 represent nonequivalent supramolecular ligand contacts, while the suffix −1 denotes the first nonisomorphic net not included in the RCSR database. The complexes were evaluated against *Plasmodium falciparum*, showing the high influence of the metal coordination in the modulation of the antiplasmodial activity relative to the free ligand, particularly against the resistant phenotype. All evaluated complexes retained inhibitory effects against both chloroquine‐sensitive and chloroquine‐resistant *P. falciparum* strains. Among the series, **C3** and **C4** emerged as the most promising candidates due to their favorable balance between antiplasmodial activity, selectivity, and low hemolytic profile. Computational analyses further revealed that modulation of the electronic properties, chemical softness, and lipophilic profiles of the complexes may contribute to their distinct biological behavior, while molecular docking suggested plausible interactions with parasite‐associated enzymatic targets.

## Graphical abstract

Four isostructural complexes (C1–C4) derived from 2‐(*tert*‐butoxy)‐6‐(1H‐imidazol‐1‐yl)pyridine with axial halides exhibit octahedral trans M(L)_4_X_2_ geometries, confirmed by X‐ray diffraction. They feature a discrete 1,6M7−1 topology, identified using ToposPro analysis. Metal coordination enhances antiplasmodial activity against *Plasmodium* *falciparum*, with complexes C3 and C4 showing the highest potency, selectivity, and minimal hemolytic activity.

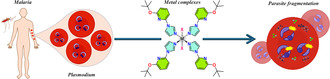

## 1 Introduction

One of the main targets of recent bioinorganic research is the *Plasmodium* parasite, the etiological agent of malaria, which remains one of the most persistent threats to global health, particularly in tropical and subtropical regions, with over 282 million cases and ≈610,000 deaths in 2024 [1, 2]. The increasing emergence of strains resistant to conventional treatments has driven the search for new compounds with potential antiplasmodial activity [3, 4, 5]. Imidazole and its derivatives are highly valued for their widespread presence in pharmaceuticals. These molecules exhibit diverse industrial applications and potential biological activity against various microorganisms. For instance, they have been used to control parasites; some benzimidazole derivatives, such as albendazole, exhibit antiparasitic activity [6, 7]. Azole‐based compounds have shown significant antiplasmodial activity, primarily attributed to their ability to inhibit hemozoin formation, a detoxification pathway used by malaria parasites to sequester free iron released from digested hemoglobin [8, 9, 10]. In addition, azoles have been reported to interfere with sterol biosynthesis by inhibiting the enzyme lanosterol 14‐α‐demethylase, potentially altering the lipid composition of the parasite's cellular membrane [9, 10, 11, 12]. Such disruption in membrane integrity and function ultimately leads to cellular dysfunction and parasite death.

Although resistance to artemisinin‐based combination therapies underscores the urgent need for antiplasmodial agents with novel mechanisms of action, the presence of a lone pair of electrons in the imidazole ring enables it to coordinate with transition metals, facilitating the formation of coordination complexes with enhanced properties for pharmacological applications and representing an important strategy for the development of new antiplasmodial agents [13, 14, 15, 16]. This enhanced biological activity is strongly influenced by the nature of the organic ligand, the metal center, and the coordination geometry, which collectively modulate physicochemical properties such as lipophilicity, reactivity, and stability under biological conditions [17, 18, 19]. Furthermore, in this area, our group has conducted various studies using azole ligands with transition metals [20, 21, 22, 23, 24, 25].

In the search for novel bioactive coordination compounds, the choice of the metal center and coordinated halide plays a decisive role in determining both the structural and biological behavior of the complexes. Chloride ligands, owing to their stronger metal–ligand bonding and higher polarity, often promote well‐defined geometries but can also increase reactivity toward biological targets, sometimes enhancing cytotoxicity [26]. In contrast, bromide ligands, characterized by greater polarizability and lipophilicity, may improve membrane permeability and favor sustained intracellular retention, thereby enhancing biological selectivity [27, 28].

In this context, this study presents the synthesis, full characterization, and antiplasmodial activity of four new octahedral coordination complexes of Co(II), Ni(II), and Cu(II) using the ligand 2‐(*tert*‐butoxy)‐6‐(1H‐imidazol‐1‐yl)pyridine (**L**).

## 2 Experimental Section

### 2.1 Synthesis of 2‐(tert‐butoxy)‐6‐(1H‐imidazol‐1‐yl)pyridine (L)

The synthesis of 2‐(*tert*‐butoxy)‐6‐(1H‐imidazol‐1‐yl)pyridine (**L**) was performed by modifying a previous report [29]. In a two‐neck Schlenk round‐bottom flask under an inert atmosphere and coupled to a condenser, 2,6‐dichloropyridine (2 g, 13.51 mmol, 1 eq), potassium tert‐butoxide (1.9 g, 16.89 mmol, 1.25 eq), and 20 mL of dry toluene were added. The mixture was heated to reflux and allowed to react for 8 h. In another Schlenk round‐bottom flask and under an inert atmosphere, imidazole (1.15 g, 16.89 mmol, 1.25 eq) and NaH (0.675 g, 16.89 mmol, 1.25 eq) in 30 mL of dry DMF at 0°C were added. The mixture was left stirring for 0.5 h at the same temperature, and the solution was transferred into the reaction in toluene with a cannula. The final mixture was heated to 160°C and left to react for 12 h. To the reaction, 100 mL of H_2_O was added, and 4 extractions of 50 mL of AcOEt were performed. The organic phase was dried with Na_2_SO_4,_ and the solvent was removed under reduced pressure. The resulting crude was purified by column chromatography using AcOEt as the mobile phase. Yield: 90%. Fourier transform infrared spectroscopy (FT‐IR) (ATR, cm^−1^): υ 2974(w), 1604(m), 1570 (m), 1446 (s), 1157 (m), 790 (m). ^1^H NMR (400 MHz, CDCl_3_): δ 8.57 (s, 1H), 7.67 (t, *J* = 8.0 Hz, 1H), 7.62 (s, 1H), 7.28 (s, 1H), 6.93 (d, *J* = 7.6 Hz, 1H), 6.64 (d, *J* = 8.2 Hz, 1H), 1.63 (s, 9H).

### 2.2 Synthesis of Complexes

The synthesis of the four complexes was carried out similarly: the ligand (**L**) was dissolved in ethanol, followed by the addition of a solution of the respective salt in a minimal amount of ethanol. The mixture was stirred under reflux at 80°C for 12 h. After this time, the solvent was removed under reduced pressure. The solid was purified by washing with heptane, and then the precipitate was dissolved in dichloromethane (DCM) and filtered. The final product was obtained after removing the DCM under reduced pressure.

#### 2.2.1 C1 [Co(Cl)_2_(L)_4_]

Blue solid. Yield: 76%. Mp: 174.30°C. FTIR (ATR, cm^−1^): υ 3148(w), 2978(w), 1605(m), 1574(m), 1273(m), 1165(s), 1011 (m). Raman (cm^−1^): 505, 471. UV–vis (λ_max_ nm) (ε, L·mol^−1^·cm^−1^): 239 (34148), 289 (44392), 575 (545), 610 (852). Conductivity (Λ_M_): 12,09. Anal. Calcd. For C_48_H_60_C_l2_CoN_12_O_4_: C: 57.72%, N: 16.83%, H: 6.05%, Found: C: 57.16%, N: 16.80%, H: 5.99%.

#### 2.2.2 C2 [Cu(Cl)_2_(L)_4_]

Blue solid. Yield: 74%. Mp: 172.40°C. FTIR (ATR, cm^−1^): υ 3132(w), 2978(w), 1609(m), 1570(m), 1269(m), 1168(s), 1011(m). Raman (cm^−1^): 402, 288. UV–vis (λ_max_ nm) (ε, L·mol^−1^·cm^−1^): 239 (28868), 289 (34732), 730 (113). Conductivity (Λ_M_): 10.95. Anal. Calcd. For C_48_H_60_Cl_2_CuN_12_O_4_: C: 57.45%, N: 16.75%, H: 6.03%, Found: C: 57.41%, N: 16.69%, H: 5.97%.

#### 2.2.3 C3 [Co(Br)_2_(L)_4_]

Pink solid. Yield: 73%. Mp: 197.50°C. FTIR (ATR, cm^−1^): υ 3144(w), 2978(w), 1605(m), 1574(m), 1273(m), 1165(s), 1011(m). Raman (cm^−1^): 477, 221. UV–vis (λ_max_ nm) (ε, L·mol^−1^·cm^−1^): 239 (32500), 289 (43376), 585 (448), 625 (769), 645 (813). Conductivity (Λ_M_): 21.40. Anal. Calcd. For C_48_H_60_Br_2_CoN_12_O_4_: C: 53.00%, N: 15.45%, H: 5.56%, Found: C: 53.01%, N: 15.37%, H: 5.56%.

#### 2.2.4 C4 [Ni(Cl)_2_(L)_4_]

Navy green solid. Yield: 74%. Mp: 237.00°C. FTIR (ATR, cm^−1^): υ 3152(w), 2978(w), 1605(m), 1574(m), 1273(m), 1165(s), 1011(m). Raman (cm^−1^): 387, 221. UV–vis (λ_max_ nm) (ε, L·mol^−1^·cm^−1^): 239 (34876), 289 (45694), 414 (66), 508 (31), 568 (28), 704 (19). Conductivity (Λ_M_): 19.11. Anal. Calcd. For C_48_H_60_C_l2_NiN_12_O_4_: C: 57.73%, N: 16.83%, H: 6.06%, Found: C: 57.72%, N: 16.73%, H: 6.04%.

### 2.3 Methodology for Crystal Growth

**C1–C3**: A saturated solution in DCM of each of these complexes was prepared. A single crystal suitable for X‐ray diffraction analysis was obtained by slow evaporation.

**C4**: Employing the slow diffusion technique from a saturated solution of **C4** in acetone: diethyl ether (3:1 mL). A single crystal suitable for X‐ray diffraction analysis was obtained.

### 2.4 Biological Evaluation

For the biological evaluation, hemolysis assays and antiplasmodial activity tests were performed using both chloroquine‐sensitive and chloroquine‐resistant *Plasmodium falciparum* strains.

### 2.5 Hemolysis Assay

Red blood cells (RBCs) were obtained by venipuncture from a healthy donor in tubes with sodium citrate. The samples were centrifuged, and plasma and leukocyte–platelet layers were removed. RBCs were washed with PBS 1X until the supernatant was clear. The suspension was adjusted to 1.6% hematocrit in McCoy's 5A medium and seeded in 96‐well plates predosed with the compounds under evaluation. Stock solutions of complexes were prepared in dimethylsulfoxide (DMSO) at a concentration of 6000 μg/mL. Working solutions at 2000 μg/mL were prepared and serially diluted (7 points). Tween‐20 (1%) served as a positive control, RBCs in PBS as a negative control, and additional wells were included to assess the effect of DMSO alone. All concentrations and controls were tested in triplicate. Plates were incubated at 37°C for 1 h. The assay was performed twice independently to confirm reproducibility.

Hemolysis was quantified using a FACSLyric flow cytometer (BD Biosciences). A gating strategy was established using RBCs lysed with 1% Tween‐20 and intact RBCs suspended in PBS 1X as reference populations. Acquired events were used to define three populations: intact erythrocytes (Gr), hemolysis debris (LGr), and background signal. This template was saved and consistently applied to all experimental samples.

### 2.6 Stability Assay in Culture Medium

The stability of the coordination complexes in culture medium was evaluated by UV–vis spectroscopy over a 96 h incubation period. Each compound was prepared at a concentration of 1000 µg/mL in complete culture medium and analyzed in triplicate under the same conditions employed for the biological assays. UV–vis spectra were recorded between 200 and 770 nm at 0, 24, 48, 72, and 96 h.

### 2.7 Drug Susceptibility Assay

The compounds were evaluated against *P. falciparum* 3D7 (chloroquine‐sensitive, NIAID, BEI Resources) and W2 (chloroquine‐resistant, NIAID, BEI Resources) strains. Parasites were maintained in continuous culture under sterile conditions and synchronized to the ring stage with sorbitol once parasitemia reached ∼6%. Cultures were adjusted to 0.25% parasitemia and seeded into predosed plates containing each compound. Antiplasmodial activity was evaluated using serial 10‐fold dilutions, with final concentrations ranging from 4 to 400,000 nM. Plates were incubated for 96 h at 37°C in a controlled gas mixture. Microscopic examination of parasite cultures was performed at 18, 24, and 46 hours postincubation. Parasite growth was assessed by fluorescence based on nucleic acid quantification with SYBR Green I. After incubation, plates were frozen at –86°C for ≥24 h, thawed at 37°C for 4 h, and 100 µL of each well was transferred to black plates with clear bottoms. Lysis buffer and SYBR Green I were added, followed by 1 h of incubation under agitation and in the dark. Mean fluorescence intensity (MFI) was measured in a multimodal plate reader (excitation: 485 nm; emission: 530 nm).

### 2.8 Statistical Analysis

MFI data were tabulated in Excel, and the mean ± SD of triplicates was calculated. The percentage of activity was determined relative to the negative control (chloroquine‐treated parasites, NC) and the growth control (infected RBCs without drug, iRBCs), according to the following equation

$$\text{Percentage}\;\mathrm{of}\;\text{activity}=100-100\times \left(\frac{\text{Average}\;\mathrm{MFI}-\mathrm{NC}}{\text{iRBCs}-\mathrm{NC}}\right)$$

where MFI is the mean fluorescence intensity of wells treated with the compound, NC is the negative control (chloroquine + parasites), and iRBC is the growth control. IC_50_ values were estimated in GraphPad Prism using a nonlinear dose–response (sigmoid) model, and curve fitting was evaluated by the coefficient of determination (*R* ^2^).

### 2.9 Computational Methodology

To accurately evaluate the electronic properties and thermodynamic stability of the coordination complexes **C1**, **C2**, **C3**, and **C4**, their initial geometries were extracted directly from the experimental X‐ray diffraction coordinates. All quantum chemistry calculations were carried out with ORCA 6.1 [30]. The geometric optimization and electronic structure refinement were performed using density functional theory with the M06‐2X hybrid meta‐GGA functional [31], which provides a robust description of noncovalent interactions and transition metal thermochemistry. The 6‐31+G(d, p) basis set was employed for all nonmetal atoms to account for electronic density polarization and diffusion, while the LANL2DZ effective core potential was applied to the Co(II), Cu(II), and Ni(II) metal centers to manage the relativistic effects of the inner electrons [32]. To simulate physiological conditions, the solvation model based on density (SMD) was implemented utilizing water as the continuous solvent [33]. Furthermore, quantum global reactivity descriptors were derived from the energy of the frontier molecular orbitals (FMO) according to Koopmans’ theorem [34]. Descriptors, such as the energy gap, chemical hardness, and chemical softness, were calculated to assess the kinetic stability and polarizability of the complexes, providing a theoretical framework to correlate their electronic structure with their enhanced lipophilic profile and passive diffusion capabilities.

To provide a quantitative assessment of the lipophilic profile and passive diffusion capabilities of the complexes, the n‐octanol/water partition coefficients (log *P*) were theoretically estimated using a thermodynamic solvation cycle. The Gibbs free energy of solvation (Δ*G*_{solv}_) was calculated for each complex in both n‐octanol and aqueous phases using the SMD continuum solvation model at the M06‐2X/6‐31+G(d, p)/LANL2DZ level of theory.

The log *P* values were derived from the difference in the free energy of transfer (ΔΔ*G*_{tr}_) between the two solvents according to the following relationships

$$\Delta \Delta G_{\left\{\mathrm{tr}\right\}}\;=\;G_{\left\{\text{octanol}\right\}}\;-\;G_{\left\{\text{water}\right\}}$$

$$\log P=\;-\Delta \Delta G_{\left\{\mathrm{tr}\right\}}/2.303\;RT$$

where *R* is the universal gas constant and *T* is the temperature (298.15 K). This approach allows a direct correlation between the electronic structure of the coordination complexes and their ability to permeate lipid bilayers, which is a critical factor for their observed in vitro antiplasmodial activity and their capacity to reach effective intracellular concentrations within the parasite.

To elucidate the atomistic details underlying the observed in vitro antiplasmodial activity, three strategic biological targets from *P. falciparum* were selected from the Protein Data Bank. The Lactate Dehydrogenase (PfLDH, PDB ID: 1T24) was selected as the primary target for azole‐based inhibitors due to its critical role in the parasite's energy metabolism. Finally, Falcipain‐2 (PfFP‐2, PDB ID: 3BPF) [35], a digestive vacuole cysteine protease central to hemoglobin degradation, was chosen to evaluate the proposed mechanism of hemozoin inhibition [36], exploiting the high affinity of transition metals for active‐site cysteine residues. Prior to the simulations, the protein structures were prepared by removing cocrystallized water molecules and heteroatoms, adding polar hydrogens, and computing Gasteiger partial charges to properly describe the electrostatic environment of each catalytic pocket [37].

Molecular docking simulations were executed using MetalDock [38], a specialized platform optimized for the prediction of binding affinities in organometallic compounds. The search space was centered on the established active sites of each respective target, and conformational sampling was guided by the Lamarckian genetic algorithm to ensure an exhaustive exploration of the binding landscape [39]. A critical step in the ligand preparation involved modifying the central metal atom identifier to the carbon atom notation within the PDBQT files. This topological adjustment ensures compatibility with the empirical scoring function and strictly preserves the bulky octahedral coordination sphere during docking. The resulting binding poses were ranked according to their binding affinity expressed in kcal/mol. The optimal conformations were then subjected to detailed visual inspection, and the network of noncovalent interactions, including π–π stacking, hydrophobic contacts, and electrostatic interactions, was characterized using SAMSON software among the active‐site residues, the equatorial organic ligands, and the metal–halogen core [40].

## 3 Results and Discussion

### 3.1 Synthesis and Characterization of Complexes

**SCHEME 1:**
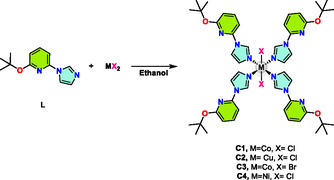
Synthesis of **C1–** **C4** complexes.

The coordination complexes were obtained from the reaction of the metal ethanolic solution with the **L** ligand. Due to the oily nature of **L**, it was readily removed from the mixture by heptane washing, and the complexes were finally isolated from the residual metal precursor by extraction with DCM followed by filtration. The four metal complexes are air‐stable and were obtained as colored solids (**C1** and **C2**: blue, **C3**: pink, **C4**: navy green) in high yield. The compound was characterized using various techniques. Elemental analysis indicated that the complexes had a ligand‐to‐metal ratio of 4:1. Therefore, four ligand (**L**) molecules are coordinated to the metal center (Scheme 1).

The molar conductivity of the complexes in acetonitrile ranged from 10 to 20 Ω^−1^ cm^2^ mol^−1^, demonstrating their nonelectrolytic behavior in solution. These values are significantly lower than those expected for 1:1 electrolytes in acetonitrile, suggesting that the bromide and chloride coligands remain coordinated to the metal center rather than dissociate into free ions. Consequently, the complexes are neutral and are represented as [M(L)_4_X_2_].

The FTIR spectra of the complexes showed absorption bands similar to those of the free ligand, which is consistent with coordination mediated by the imidazole nitrogen atom, leaving most of the ligand framework virtually unchanged [41, 42, 43]. The band assigned to the C=N stretching vibration, observed at 1570 cm^−1^ in the free ligand, was shifted to 1574 cm^−1^ in complexes **C1**, **C3**, and **C4** (Table S1). This shift toward higher wavenumbers can be attributed to the coordination of the imidazole nitrogen atom with the metal center, which alters the distribution of electronic density in the conjugated system [44, 45]. As a result, the electronic density over the C=N bond decreases, leading to a partial increase in its double‐bond character and, consequently, to an increase in its vibrational frequency [46, 47]. Similarly, the band associated with the C–N vibration of the imidazole ring in the free ligand is observed between 1165 and 1168 cm^−1^ and shifts to 1157 cm^−1^ in the complexes, supporting the involvement of the imidazole group in the coordination [48]**.** Small shifts were also observed in the vibrations of the aromatic C=C, the pyridine ring, and the C–O–C following coordination, indicating slight perturbations in the ligand's conjugated electronic system upon binding to the metal [49, 50, 51]. Furthermore, FTIR spectroscopy confirmed the solid‐state stability of the ligand and the synthesized complexes, revealing that the vibrational bands associated with the ligands and the metal center persisted for up to 30 days, thereby corroborating the structural stability of the system (Figures S6, S14, S22, and S30).

The Raman spectra allowed the assignment of the metal–ligand (M–N) and metal–halide (M–X) vibrations. For **C1** and **C3**, the bands at 505 and 477 cm^−1^ were attributed to Co–N vibrations, while for **C2** and **C4**, the M–N bands appeared at 402 and 387 cm^−1^, respectively (Table S1) [52, 53]. Additionally, the M–X vibrations for **C1**–**C4** were observed at 471, 288, 221, and 221 cm^−1^, respectively (Figures S7, S15, S23, and S31) [21, 23, 24, 52, 53, 54]. The lower frequencies observed in complexes containing bromide as a coligand are consistent with bromide's higher atomic mass compared to chloride, which results in lower vibrational frequencies in the corresponding M–Br bonds [23, 52]. The shifts observed in both FTIR and Raman spectra are consistent with those reported for related transition metal imidazole complexes, particularly for the vibrations associated with the C=N and C–N bonds involved in coordination through the imidazole nitrogen atom [55, 56, 57, 58, 59].

To determine the behavior of the complexes in solution, UV–vis analysis was performed on DCM solutions of the complexes (Figures S3, S8, S9, S16, S17, S24, S25, S32, and S33). The UV–vis spectrum of L shows bands in the range of 243 and 289 nm, which can be assigned to the n‐π* transitions (243 nm) from the lone pair electrons of nitrogen in imidazole and pyridine, and to the π‐π* transitions (289 nm) of the conjugated systems [29]. For the complexes, a hypsochromic shift in the n‐π* signal was observed at 239 nm, which can be attributed to the lone pair coordination to the metal center (Table S2). Regarding the complexes, in the UV–vis spectrum of **C1**, the bands observed at 575 and 610 nm can be attributed to the *d–d* transitions ^4^T_1g_(F) → ^4^A_2g_(F) and ^4^T_1g_(F) → ^4^T_1g_(*P*), characteristic of octahedral Co(II) complexes [60, 61, 62]. For **C2**, the broad band centered at 730 nm can be assigned to ligand‐field *d–d* transitions characteristic of distorted octahedral Cu(II) complexes [63, 64]. In **C3**, the bands observed at 585, 625, and 645 nm can be attributed to the *d–d* transitions characteristic of octahedral Co(II) complexes, ^4^T_1g_(F) → ^4^T_2g_(F), ^4^T_1g_(F) → ^4^A_2g_(F), and ^4^T_1g_(F) → ^4^T_1g_(P) [65, 66]. The slight shifts relative to **C1** may be associated with the presence of the bromide ligand, which displaces the ligand field due to its position in the spectrochemical series relative to the chloro ligand [67]. Finally, for **C4**, the bands *d–d* were observed at 508, 568, and 704 nm, corresponding to the transitions ^3^A_2g_(F) → ^3^T_2g_(F), ^3^A_2g_(F) → ^3^T_1g_(F), and ^3^A_2g_(F) → ^3^T_1g_(P), characteristic of octahedral Ni(II) complexes [68]. It is possible to attribute the band at 414 nm to charge transfer activity [63, 67, 69, 70]. For **C1–** **C3**, this band is perhaps present but could be overlaped by the UV‐region ones. (Figures S8, S9, S16, S17, S24, S25) [60, 63, 67, 69, 70]. In addition, the stability of the complexes in solution was evaluated using UV–vis spectroscopy, which showed that the characteristic signals remained unchanged for at least 5 days, indicating stability in solution (Figures S10, S18, S26, and S34).

The free ligand exhibits fluorescence emission centered at 390 nm, albeit with a relatively low intensity (Figure S4). However, upon formation of the coordination complexes, complete quenching of ligand fluorescence was observed for all complexes (Figures S11, S19, S27, and S35). The suppression of the emissive phenomenon can be attributed to charge–transfer or deactivation processes at the metal center, which are common in systems where the metal facilitates alternative energy‐dissipation pathways, such as electron transfer or intersystem conversion [71, 72].

To determine the thermal stability of the complexes, thermogravimetric analysis was carried out. All results are proposed based on probable mass losses because the decomposition products could not be detected. As can be observed in Table S3, all the complexes presented a significant initial loss (177.06°C to 224.46°C) (Figures S12, S20, S28, and S36). For C1, it was 50.83%, attributable to the loss of two L molecules and two Cl^−^ coligands (theoretical loss of 50.60%). For the other complexes, a similar behavior was observed, with a mass loss corresponding to the loss of two L molecules and only one of the coligands during this first mass loss. These losses are 45.75%, 48.53%, and 46.88%, respectively, for C2, C3, and C4 (In the same way, theoretical losses are 46.83%, 47.29%, and 47.06%, respectively). As the temperature increases, additional mass losses are observed, corresponding to the thermal decomposition of the ligands. Also, this analysis revealed that C4 exhibits the greatest thermal stability among the systems studied, with a decomposition onset temperature of 205.99°C. This behavior indicates that these compounds are thermally stable, which may reflect their robust solid‐state structures.

### 3.2 Crystallographic Analysis

A search of the Cambridge Structural Database found that no crystal structures of the 2‐(*tert*‐butoxy)‐6‐(1H‐imidazol‐1‐yl)pyridine ligand with Co, Cu, or Ni atoms have been reported (date of search: August 2025). Considering this finding, the crystal structures of these coordination complexes correspond to the first structural description for compounds with such chemical composition.

The crystal structures of **C1, C2**, **C3**, and **C4** were determined using the single‐crystal X‐ray diffraction technique. Table S4 shows the crystal data and additional experimental details. From these results, it can be deduced that all compounds crystallize in the same triclinic *P*−1 space group, with similar unit cell parameters. However, the unit cell volumes differ among compounds due to their distinct atomic compositions, with **C3** having the largest calculated unit cell volume due to the coordination of Br atoms. In all cases, the molecules have an inversion center in the metal atom, which means that the number of symmetry‐independent formula units within the asymmetric unit has a value of Z′ = 0.5. However, the number of formula units within the complete unit cell in all cases has a value of Z = 1, which means one molecule for the unit cell and half a molecule for the asymmetric unit in all compounds.

**FIGURE 1:**
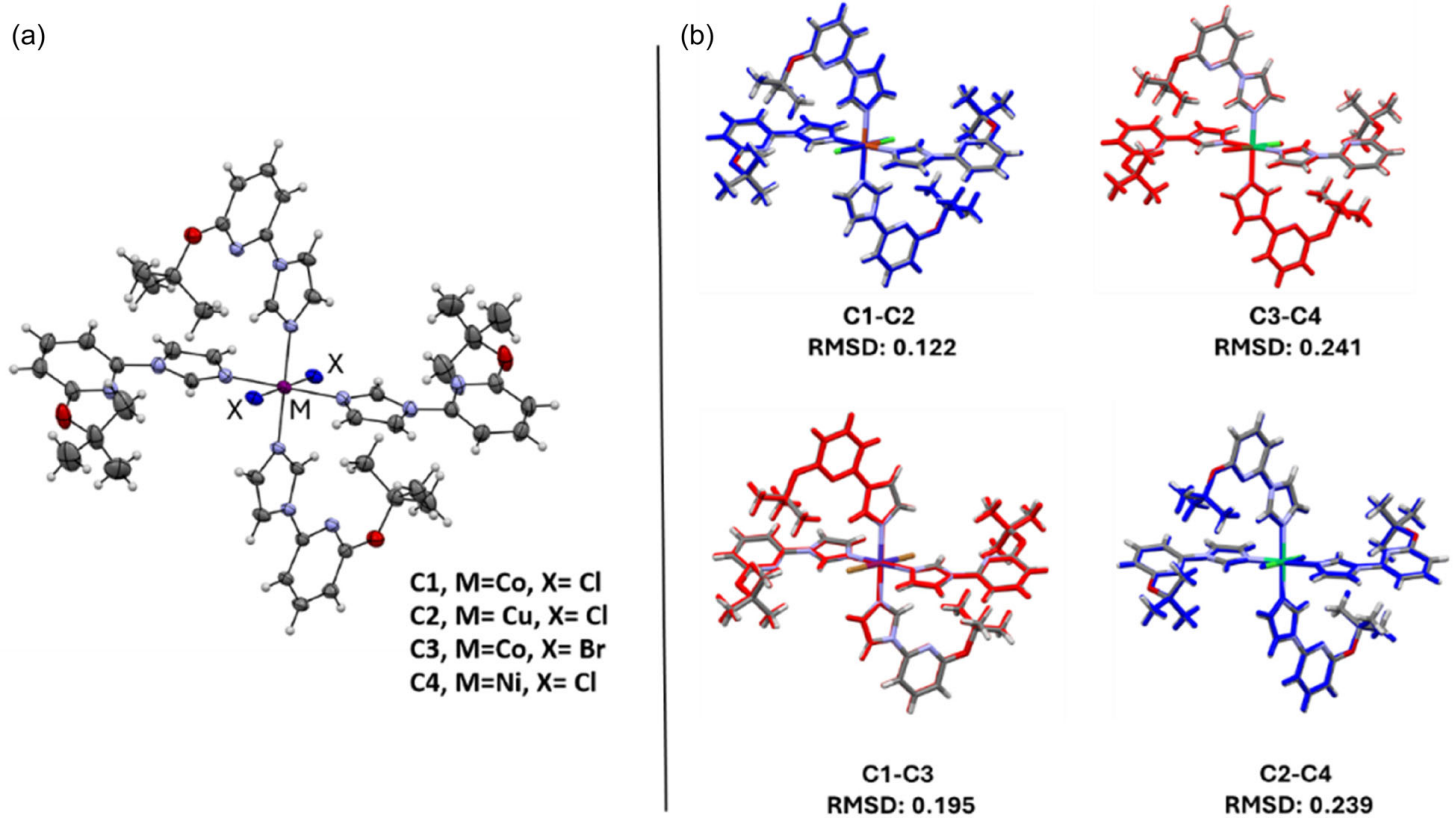
(a) Molecular structure of **C1** **–C4** with anisotropic thermal vibration ellipsoids drawn at the 30% probability level. H atoms are shown as spheres of arbitrary radii. (b) Molecular overlay showing that **C1** **–C4** are isostructural.

The molecular structures of **C1–** **C4** are characterized by an octahedral coordination of the metal center, with 4 molecules of 2‐(*tert*‐butoxy)‐6‐(1H‐imidazol‐1‐yl)pyridine ligand in equatorial coordination and 2 halogen atoms in axial coordination Figure 1a. In all cases, the N–M and X–M (M = Cu, Co, Ni; X = Cl, Br) bond distances differ despite structural similarities (Table 1). The oxidation states of the metal centers are 2+ as predicted by the valence bond model [73], and the ionic radii can be distributed as Co^2+^ > Cu^2+^ > Ni^2+^ according to the effective ionic radii for 6‐coordinated ions [74]. Consistent with this trend, the N–M distances are longer in **C1** and **C3**. However, the longer N–M distances in **C4** compared to **C2** are due to a pronounced Jahn–Teller effect in **C2**, with Cl–Cu distances longer than in other compounds, allowing closer interaction between the Cu atoms and the equatorial ligands. The octahedral volumes and quadratic elongations are 15.625 A^3^/1.017, 15.685 A^3^/1.063, 16.752 A^3^/1.032, and 14.820 A^3^/1.016 for **C1**, **C2**, **C3**, and **C4**, respectively. The higher octahedral volume corresponds to **C3** due to the Br‐coordination, and the distorted octahedra correspond to **C2** due to Jahn–Teller effects.

**TABLE 1:** N–M and X–M (M = Cu, Co, Ni) bond distances for **1, 2, 3,** and **4**.

	C1 (M = Co; X = Cl)	C2 (M = Cu; X = Cl)	C3 (M = Co; X = Br)	C4 (M = Ni; X = Cl)
N(A)–M	2.1262(16)	2.032(2)	2.1562(14)	2.0923(15)
N(B)–M	2.1542(14)	2.006(2)	2.1165(16)	2.1125(14)
X–M	2.5638(5)	2.8884(8)	2.7587(3)	2.5182(5)

**FIGURE 2:**
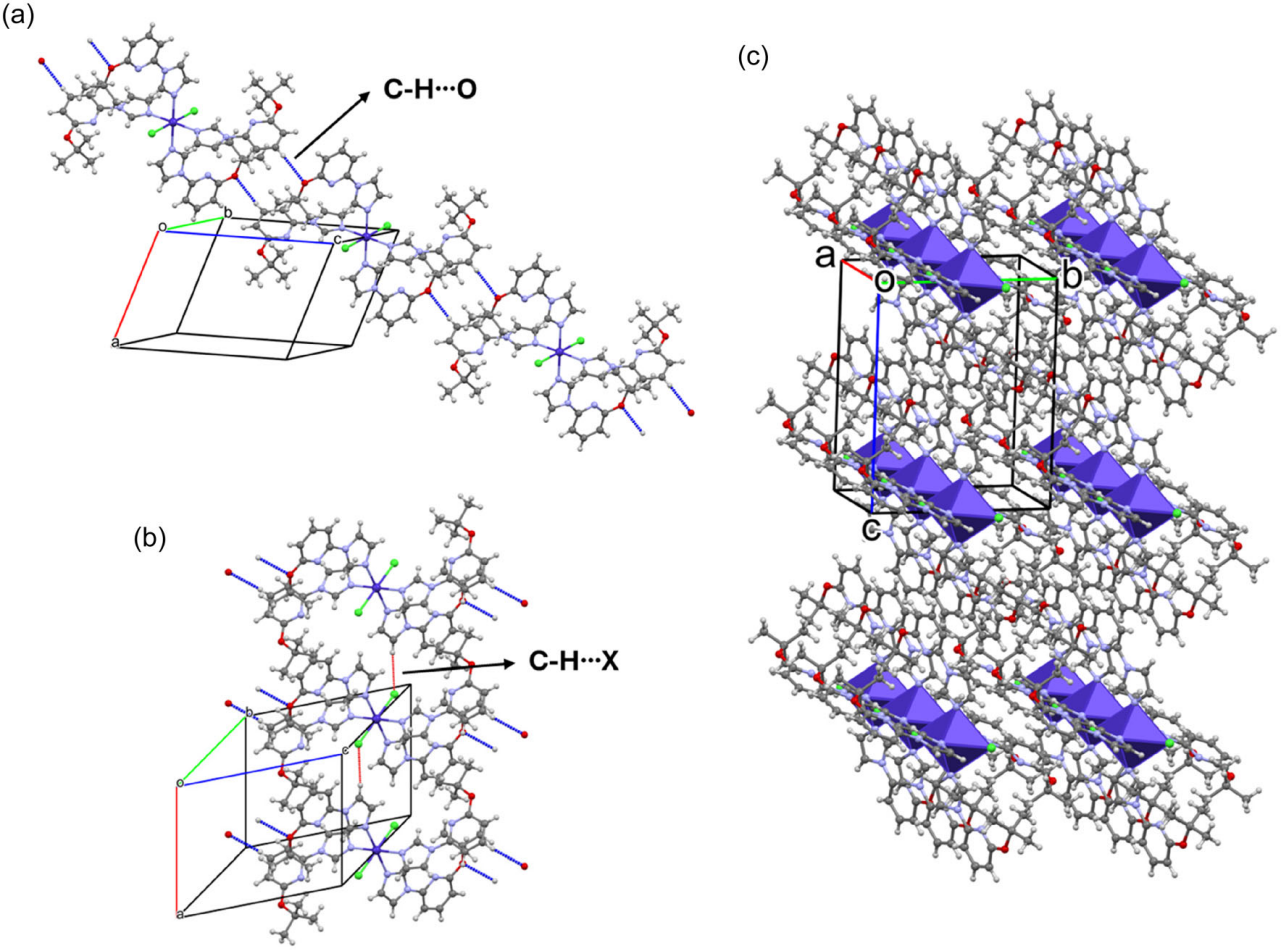
Crystal structure of **C1–** **C4** showing (a) C–H···O and (b) C–H···X hydrogen bonds. (c) Molecular packing shows the polyhedral structural distribution.

From a general perspective, the four compounds can be understood as isostructural systems from both intermolecular and intramolecular perspectives (Figure 1b and Table S4). In the supramolecular structures of **C1** **–C4**, C–H···O hydrogen bonds connect molecules in chains through the pyridine fragment in one molecule and the nonbonding electrons of the *tert*‐butoxy moiety from the neighboring molecule (Figure 2a and Table 2). These hydrogen bonds are the shortest interactions in the packing due to the pyridine ring's acidic tendency. In this context, the ligand molecules are determinants of crystal formation, which explains the isostructural behavior. Two neighboring chains are connected by longer C–H···X (X = Cl, Br), contributing to the packing along the [100] direction (Figure 2b and Table 2). The supramolecular structures in **C1–** **C4** allow the octahedra to form columns oriented along the [100] direction with distances between them of 9.113, 11.141, and 14.322 Å along *a*, *b*, and c axes, respectively (Figure 2c).

**TABLE 2:** Hydrogen‐bond geometry (Å, °) for compounds **C1, C2, C3, C4**.

*D*—H···*A*	*D*—H	H···*A*	*D*···*A*	*D*—H···*A*
**C1**				
C6*B*—H6*B*···O1*A* a	0.93	2.53	3.349(3)	147
C2*A*—H2*A*···Cl1^b^	0.93	2.88	3.7685 (18)	161
**C2**				
C6*A*—H6*A*···O1*B* c	0.93	2.53	3.350(5)	148
C2*B*—H2*B*···Cl1^d^	0.93	2.72	3.621 (3)	163
**C3**				
C6*A*—H6*A*···O1*B* e	0.93	2.53	3.364(3)	149
C2*B*—H2*B*···Br1^f^	0.93	2.93	3.8098 (18)	159
**C4**				
C6*B*—H6*B*···O1*A* g	0.93	2.53	3.352(3)	147
C2*A*—H2*A*···Cl1^h^	0.93	2.89	3.7787 (18)	160

Symmetry codes:

a −1+x,−1+y,‐1+z.

b −*x* + 1, −*y* + 1, −*z* + 2.

c −1+x,‐1+y,‐1+z.

d *x* + 1, y, z;

e 1+x, 1+y, 1+z.

f −x−1, −y, −z.

g −x,−y, 1−z.

h −x, −*y* + 1, −*z* + 2.

Hydrogen bonds and other interactions can be observed using Hirshfeld surface (Hs) analysis with the *CrystalExplorer* software [75], which allows the visualization of interactions within the crystal structure. Assigning *d*_e_ and *d*_i_ as the external and internal distances from a point on the Hirshfeld surface to the nearest atoms, and normalizing these values by the van der Waals (vdW) radii of the corresponding atoms, gives rise to the *d*_norm_ surface. Interactions shorter than the sum of the vdW radii give negative dnorm values and appear as red spots on the surface. Contacts close to the vdW distance are shown in white, whereas longer contacts are displayed in blue and the two atoms which yield negative values, highlighted on the surface as red spots. Interactions close to the vdW limit are shown in white, and those longer than the sum of the vdW radii are displayed in blue on the surface.

**FIGURE 3:**
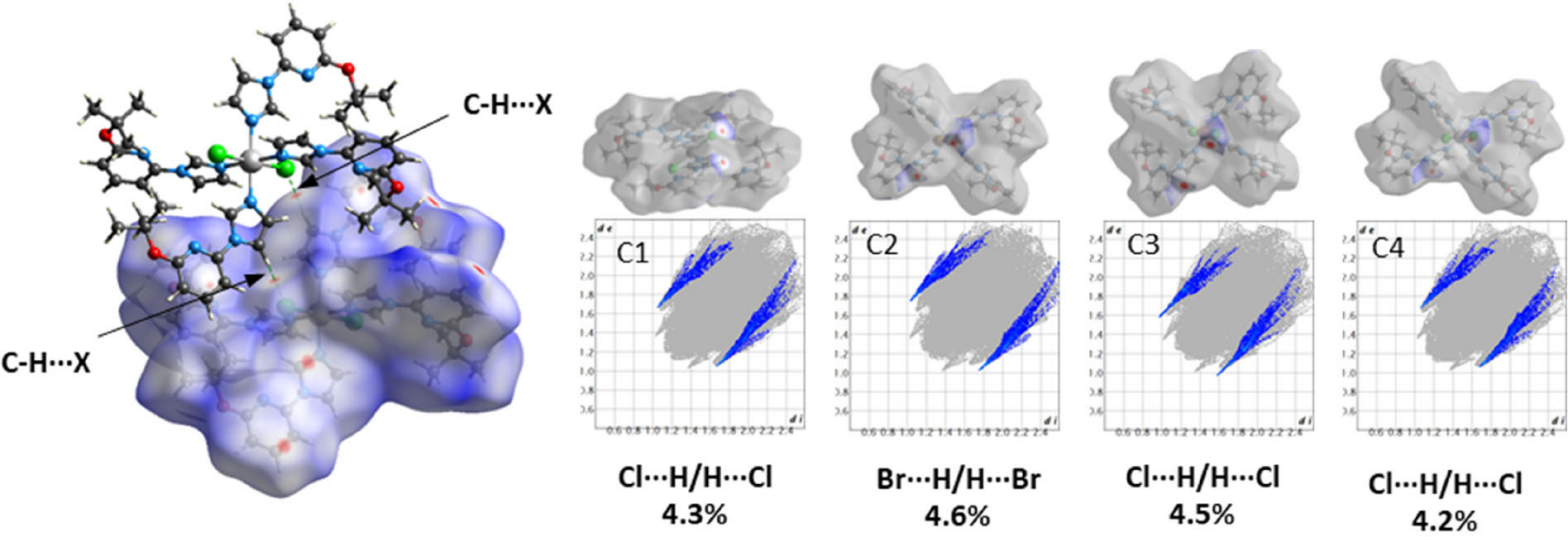
Hs maps and 2D fingerprint plots for **C1**–**C4**, showing the C–H···X interactions (X = Cl, Br) with their respective surface contributions. The bottom image highlights halogen contacts (C–H···X).

In Figure 3, the Hs mapped over *d*_norm_ is shown for compounds **C1**–**C4**. The importance of the halogen atom in the supramolecular structure is evidenced on the surface by the red spots. In this sense, H···X/X···H interactions (X = Cl for **C1**, **C2**, and **C4**; X = Br for **C3**) account for 4.3%, 4.5%, 4.6%, and 4.2% of the total Hs surface for **C1**, **C2**, **C3**, and **C4**, respectively, which corresponds to the nonconventional C–H···Cl hydrogen bonds mentioned in Table 2 for each compound.

**FIGURE 4:**
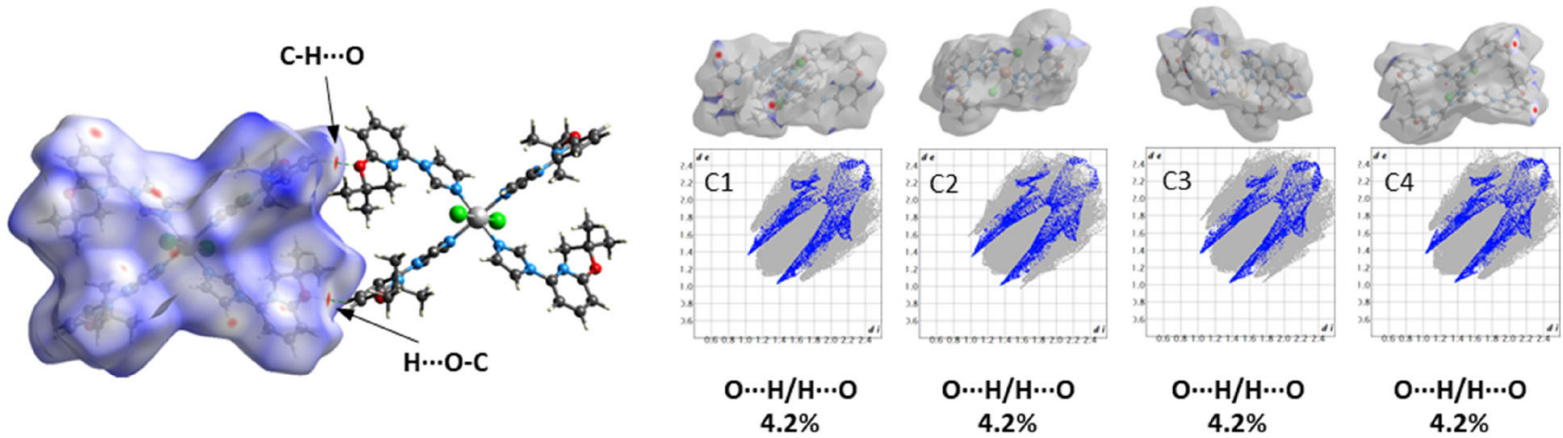
Hss and fingerprint plots for C1–C4, showing C–H···O interactions (4.2%). Below is a representation of the surface and corresponding crystal contacts.

Other nonconventional hydrogen‐bonding interactions are C–H···O (Figure 4), which account for 4.2% of the total Hs for all molecules (**C1**–**C4**), as shown in Table 3. The H···H interactions represent 63.8, 63.3, 64.0, and 63.8% of the Hs surface for molecules **C1–C4**, respectively, indicating that dispersion forces and hydrophobic contacts govern the packing and favor the formation of a compact supramolecular framework.

**TABLE 3:** Solid angle (SA), centroid–metal distance (R), and symmetry operations for the nodes in complexes **C1–C4. ZA1** and **ZA2** correspond to the ligand, ZB1 to the halogen, and ZC to the metal.

Complex	Node 1	Node 2	SA	R	Symetry code
**C1**	ZC	ZA1^a^	18,2	5,763	(0,0,–1)
		ZA1	18,2	5,763	(−x,−y,−z) (−1,0,0)
		ZA2^a^	18,5	5,543	(−x,−y,−z) (−1,0,−1)
		ZA2	18,5	5,543	—
		ZB1^a^	13,3	2,564	(0,0,–1)
		ZB1	13,3	2,564	(−x,−y,−z) (−1,0,0)
**C2**	ZC	ZA1^a^	19,8	5,659	(−x,−y,−z)
		ZA1	19,8	5,659	—
		ZA2^a^	20,3	5,499	(−x,−y,−z)
		ZA2	20,3	5,499	—
		ZB1^a^	9,9	2,888	(−x,−y,−z)
		ZB1	9,9	2,888	—
**C3**	ZC	ZA1^a^	18,8	5,744	(−1,0,0)
		ZA1	18,8	5,744	(−x,−y,−z) (0,–1,−1)
		ZA2^a^	19,3	5,558	(−x,−y,−z)
		ZA2	19,3	5,558	(−1,−1,−1)
		ZB1^a^	11,9	2,759	(−x,−y,−z) (0,–1,−1)
		ZB1	11,9	2,759	(−1,0,0)
**C4**	ZC	ZA1^a^	18	5,722	(0,0,–1)
		ZA1	18	5,722	(−x,−y,−z)
		ZA2^a^	18,4	5,529	(0,0,–1)
		ZA2	18,4	5,529	(−x,−y,−z)
		ZB1	13,3	2,518	(−x,−y,−z)(0,0,–1)
		ZB1	13,3	2,518	—

a Distinguishes symmetry‐related equivalent nodes.

### 3.3 Topological Analysis

**FIGURE 5:**
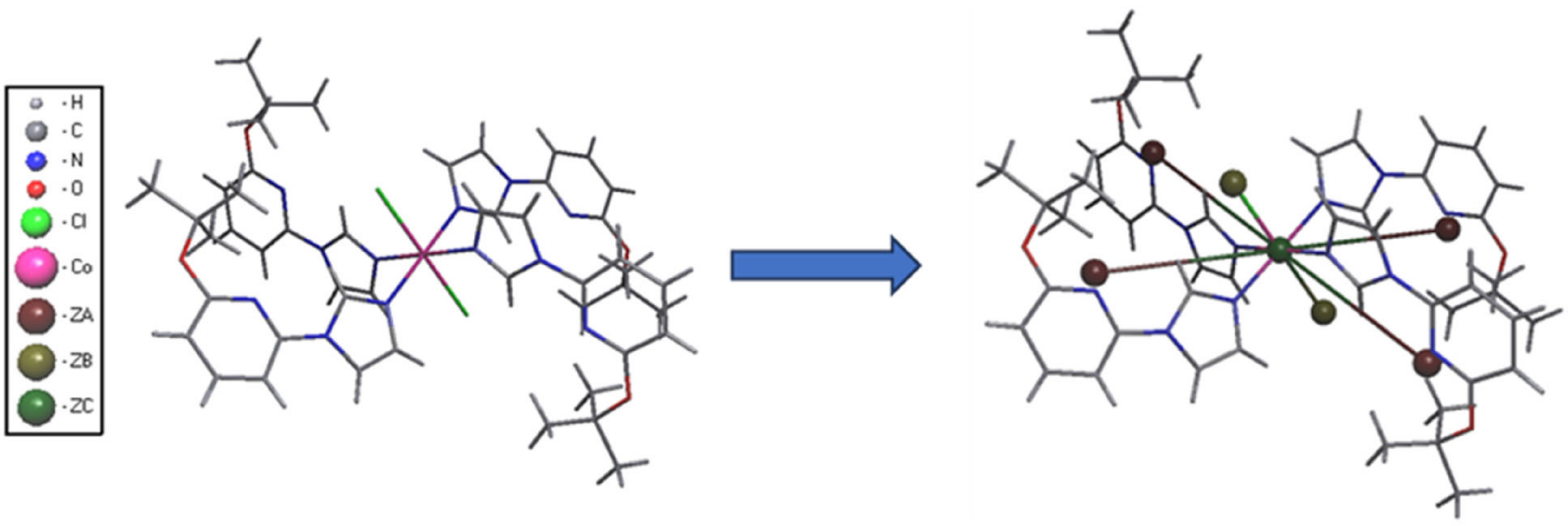
The structural simplification process in topological analysis with ToposPro involves transforming the crystallographic structure into a nodal representation. In this, **ZA** denotes the ligand, **ZB** the halogens, and **ZC** the metal atom.

The topological analysis of the crystal structures was performed using the ToposPro software package [76]. The atomic decomposition algorithm was applied to simplify the networks, enabling the identification of coordination nodes and their connectivity [77]. The resulting topological classification was obtained using the TopCryst module, which assigns codes based on the net's abstract connectivity [76, 78, 79, 80]. The metal and halogen atoms are treated as irreducible nodes, as they represent unique atoms, whereas for the ligand, the software calculates its center of gravity (Figure 5). For clarity, the notation ZA is used to refer to the ligand 2‐(*tert*‐butoxy)‐6‐(1H‐imidazol‐1‐yl)pyridine, ZB to the halogens, and ZC to the metal atoms.

In the topological analysis, the organic ligand is represented by its centroid. However, due to symmetry operations within the unit cell, this centroid does not always occupy a single equivalent position; instead, it may split into two topologically distinct variants. For this reason, two nodes are distinguished, designated as ZA1 and ZA2, which reflect the different orientations and supramolecular environments of the same ligand. In all four complexes, ZA1 and ZA2 correspond to two distinct centroid nodes associated with the same organic ligand (2‐(tert‐butoxy)‐6‐(1H‐imidazol‐1‐yl)pyridine). ToposPro analysis reveals that this ligand is involved in distinct supramolecular interactions.

In **C2**, where the metal–halogen interaction is weaker, ZA2 consistently exhibits a higher solid angle (SA) value and a shorter centroid–metal distance (R) compared to ZA1. This indicates that ZA2 occupies a geometric position that brings more ligand mass and volume into the metal environment, whereas ZA1 tends to adopt a more distant or less‐overlapping orientation.

This behavior is consistent with crystallographic analysis. For example, in **C2**, the previously mentioned Jahn–Teller effect elongates the axial Cu–Cl bonds, weakening that interaction and causing the equatorial environment, and therefore the ZA2 centroid, to move closer, which explains its higher SA. In contrast, in **C1** and **C4**, where the M–Cl interactions are stronger and shorter, the centroids remain slightly farther apart (lower SA). In **C3**, the larger size of Br expands the octahedron and generates intermediate values for ZA1 and ZA2.

**FIGURE 6:**
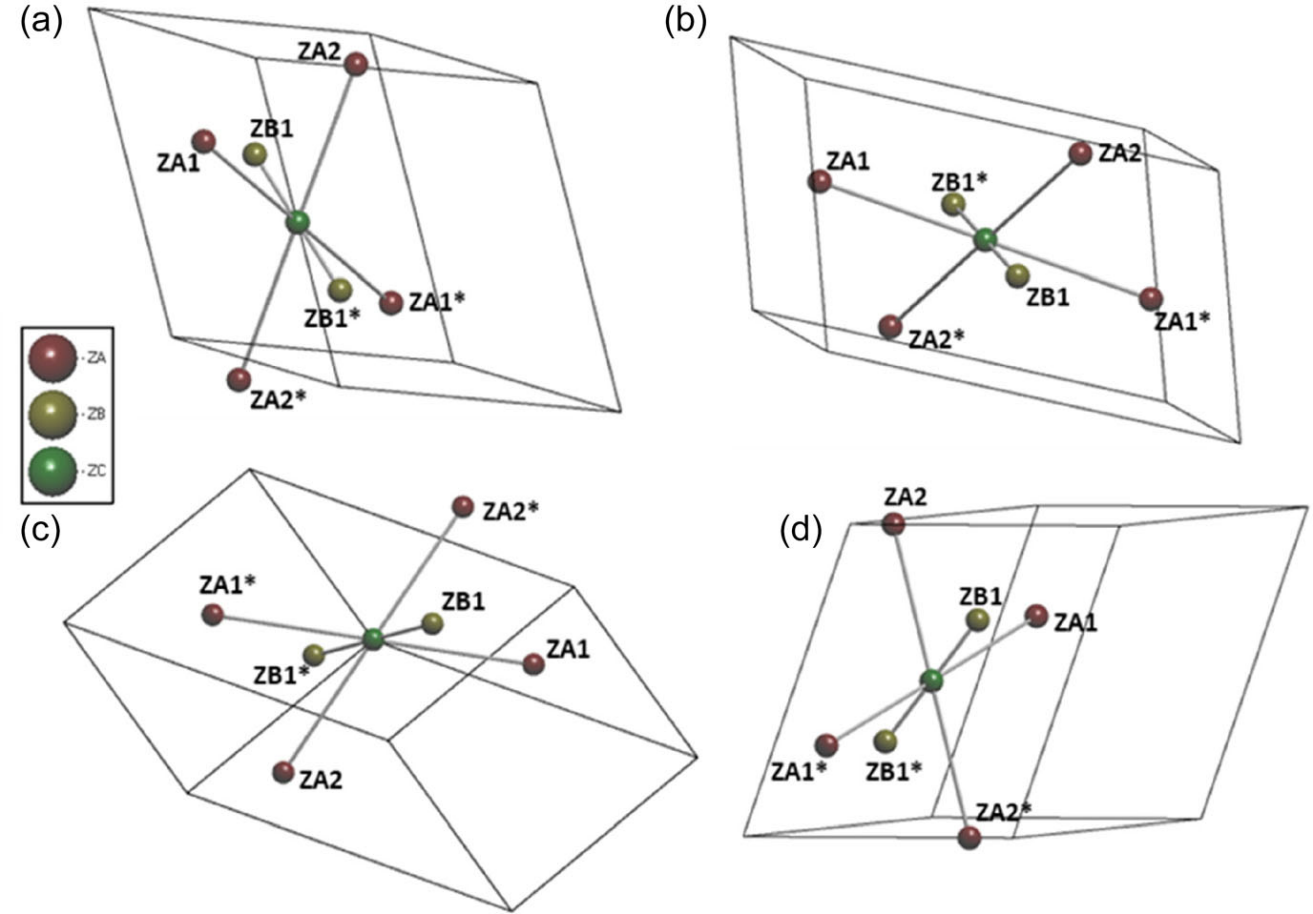
Schematic representation of the topological nodes identified through ToposPro analysis for complexes (a) **C1**, (b) **C2**, (c) **C3**, and (d) **C4**. The asterisk (*) distinguishes positions related by symmetry operations.

Although the four complexes are isostructural, the symmetry operations assigned to each node (Figure 6) are not identical among them, since they depend on the combination of the central metal and the coordinated halogen. These variations in local geometry (such as halogen size or metal‐induced distortions) affect how equivalent positions are generated within the unit cell, which explains why each complex exhibits a specific set of symmetry operations despite preserving the same structural framework.

From this nodal description, the topological analysis was complemented by the classification generated by TopCryst, which assigns codes based on the network's abstract connectivity. For all studied complexes, the resulting topology corresponds to designation 1,6M7−1, indicating two nonequivalent node types with coordination numbers of 1 and 6, respectively. The letter M denotes a 0‐periodic network, meaning a molecular net. The descriptor “7” refers to the number of nodes in the minimal representation of the SBU, while the suffix −1 indicates the first nonisomorphic net identified with this coordination sequence, meaning it is not listed in the RCSR database. This topology is depicted as a structure with a hexacoordinated central node connected to terminal monocoordinated nodes, which could serve as a basis for future supramolecular self‐assembly processes.

### 3.4 Evaluation of Biological Activity

**FIGURE 7:**
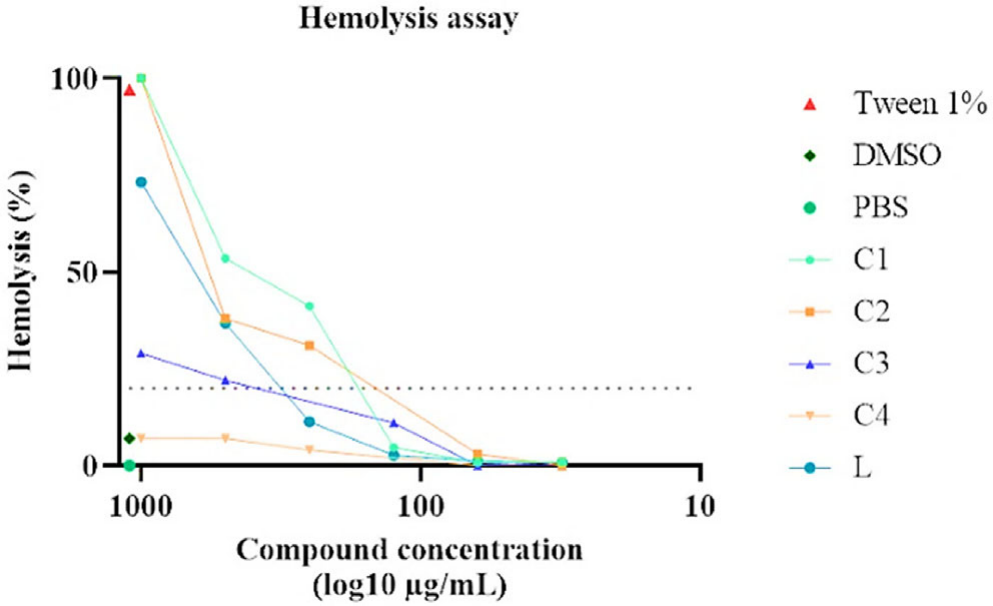
Concentration‐dependent hemolysis induced by coordination complexes **C1**–**C4** and ligand L in human erythrocytes determined by flow cytometry. PBS, DMSO, and Tween (1%) were included as negative, vehicle, and positive controls, respectively. The dashed line indicates the 20% hemolysis threshold used in this study for comparative toxicity analysis [81].

All compounds exhibited low hemolytic activity across the evaluated concentration range. Complexes **C3, C4**, and the free ligand (**L**) maintained hemolysis levels below 20% at concentrations up to 500 µg/mL, whereas **C1** and **C2** only remained below this threshold at concentrations of 250 µg/mL or lower (Figure 7). Overall, the evaluated complexes displayed acceptable erythrocyte compatibility, consistent with previous reports for related coordination compounds [82, 83, 84].

Given that none of the evaluated complexes induced 20% hemolysis within the tested concentration range, HC_20_ values could not be determined; therefore, minimum selectivity indices (SI_min_) were estimated. Among the evaluated compounds, **C4** exhibited the highest selectivity indices in both parasite strains, while **C3** displayed the lowest IC_50_ value against the chloroquine‐sensitive 3D7 strain, indicating a favorable balance between antiplasmodial potency and host‐cell compatibility (Table 4, Figure S37). In addition, the evaluated complexes demonstrated stability over time under the experimental conditions, and the corresponding data are presented in Figure S38.

**TABLE 4:** Antiplasmodial activity (*P. falciparum* 3D7) and minimum selectivity indices of the evaluated compounds.

Compound	IC_50_ 3D7	Relative IC_50_ to L	SI_mi__n_ a 3D7	IC_50_ W2	Relative IC_50_ to L	SI_mi__n_ a W2
	µM			µM		
**L**	3.57	1	>644.9	25.35	1.00	>90.8
**C1**	7.11	1.99	>17.6	2.51	0.10	>49.9
**C2**	5.01	1.40	>12.4	1.28	0.05	>48.7
**C3**	1.25	0.35	>92.3	2.16	0.09	>53.2
**C4**	2.63	0.74	>380.7	2.83	0.11	>353.7
**Chloroquine**	3.01		—	98.3		—

a Selectivity indices (SI_mi__n_) were estimated using the highest compound concentration (µg/mL) that produced less than 20% hemolysis, as HC_50_ values could not be determined within the tested range. Chloroquine was included as a reference antimalarial compound.

The antiplasmodial evaluation revealed distinct activity profiles among the tested compounds. Complex **C3** exhibited the lowest IC_50_ value against the 3D7 strain (1.25 µM), whereas **C4** showed comparable IC_50_ values against both 3D7 and W2 strains, suggesting a more consistent inhibitory profile across parasite strains. Notably, both **C3** and **C4** displayed lower IC_50_ values than the free ligand (**L**) against the 3D7 strain, while all evaluated metal complexes exhibited lower IC_50_ values than **L** against the chloroquine‐resistant W2 strain. These findings suggest that metal coordination modulates the biological behavior of the evaluated compounds and may induce inhibitory activity against resistant *P. falciparum* parasites.

The presence of imidazole moieties in these complexes may further enhance their antiplasmodial activity, as imidazole‐based derivatives have been associated with improved interactions with parasite targets [85]. In this context, the combination of an imidazole‐based scaffold with transition‐metal coordination may modulate the physicochemical and biological properties of the evaluated compounds, contributing to the emerging structure–activity relationships observed in this series.

The distinct antiplasmodial profiles observed for the metal complexes relative to the free ligand support the concept that coordination to transition metals increases biological efficacy, which is in In particular, the lower IC_50_ values observed for complexes line with Overtone's concept and Tweedy's chelation theory [86]. **C3** and **C4** against the **3D7** strains can be attributed to their stability and moderated redox profiles, which favor intracellular accumulation and interference with heme detoxification without triggering antioxidant resistance mechanisms [87, 88, 89, 90]. Variations in biological behavior were associated not only with the identity of the coordinated metal—Co(II) in **C1** and **C3**, Cu(II) in **C2**, and Ni(II) in **C4**—but also with the nature of the halide substituent. Whereas **C1**, **C2**, and **C4** contain chloride, **C3** incorporates bromide, and this substitution may influence key parameters such as electronic polarizability, lipophilic behavior, and potential membrane interactions. In agreement with the computational analyses (next section), the higher polarizability associated with Br^‐^ may contribute to a more favorable balance between membrane permeability and intracellular accumulation, which could partially explain the lower IC_50_ value observed for **C3** relative to its chloride analog **C1**. Similar trends have been reported for tetraazamacrocyclic and quinone‐based metal complexes, in which metal coordination influenced antiplasmodial behavior by modulating redox and physicochemical properties [91, 92, 93, 94]. Overall, these findings suggest that both the metal center and the coordinated halide contribute to the inhibitory profiles of these complexes while maintaining acceptable erythrocyte compatibility.

Consistent with previous reports involving Co(II), Cu(II), and Ni(II)‐based antimalarial coordination compounds [95], complexes containing chloroquine and mefloquine ligands have shown improved efficacy and reduced toxicity, particularly for Co(II) derivatives compared with Ni(II) analogues, while coordination of quinoline‐based antimalarials to transition metals such as Cu(II), Co(II), and Ni(II) has been widely explored to enhance biological performance. In line with these observations, the present results indicate that the coordinated metal modulates activity, with Cu(II)‐ and Co(II)‐containing complexes exhibiting notable activity, whereas Ni(II) coordination provides a more balanced efficacy–safety profile [96].

**FIGURE 8:**
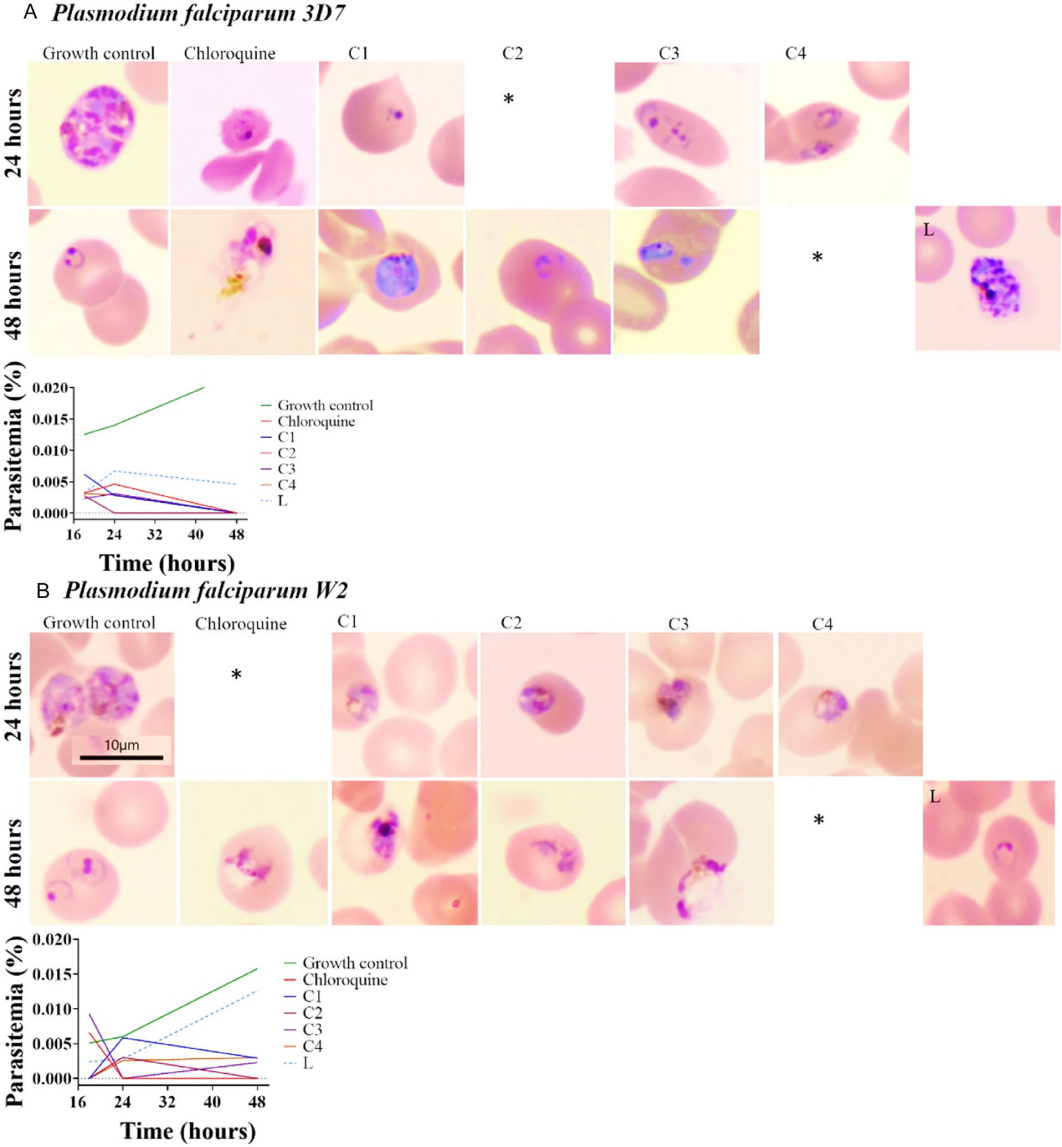
Inhibition of growth in synchronized *P. falciparum* 3D7 and W2 cultures treated with chloroquine, ligand (**L**), and metal complexes (**C1–C4**) at 24 and 48 h after culture synchronization at ring/trophozoite stage. Representative Giemsa‐stained micrographs of control and treated *P. falciparum* cultures and parasitemia kinetics are shown. *P. falciparum‐infected erythrocytes were not found in these cultures. Metal complexes induced morphological alterations and reduced parasite growth compared with controls. Scale bar = 10 µm.

Consistent with the inhibitory profiles observed in the antiplasmodial assays, morphological analysis of synchronized parasite cultures further supported the biological effects of the evaluated complexes. Treatment with **C2, C3**, and **C4** induced evident alterations in parasite morphology, including reduced cytoplasmic integrity, abnormal parasite forms, and cellular fragmentation, accompanied by decreased parasitemia relative to the growth control (Figure 8). These observations were particularly evident at 48 h and were consistent with the lower IC_50_ values observed for these complexes, reinforcing the biological relevance of their antiplasmodial effects.

### 3.5 Computational Analysis of Electronic Reactivity and Lipophilic Properties

#### 3.5.1 Electronic Parameters

The electronic parameters, calculated at the M06‐2X/6‐31+G(d, p)/LANL2DZ level of theory, revealed distinct variations (Table 5). Complex **C1** (Co–Cl) exhibited the widest energy gap (ΔE = 3.72 eV) and highest chemical hardness (1.86 eV), suggesting greater electronic stability. In contrast, complex **C2** (Cu–Cl) displayed the narrowest gap (1.36 eV) and the highest chemical softness (0.74 eV^−1^), reflecting the pronounced Jahn–Teller distortion of the Cu(II) core and its high intrinsic reactivity. Complex **C4** (Ni–Cl) exhibited an intermediate profile, with a gap of 2.02 eV and a softness of 0.50 eV^−1^, which may contribute to its more consistent antiplasmodial activity across both parasite strains.

**TABLE 5:** Global electronic parameters of the complexes calculated at the M06‐2X/6‐31+G(d, p)/LANL2DZ level of theory.

Complex	Metal–Halogen	E_{HOMO}, eV	E_{LUMO} (eV)	Gap Δ*E*, eV	Hardness η, eV	Softness S, eV^−1^	IC_50_ 3D7, μM*
**C1**	Co(II)–Cl	−8.13	−4.40	3.72	1.86	0.27	7.11
**C2**	Cu(II)–Cl	−7.93	−6.58	1.36	0.68	0.74	5.01
**C3**	Co(II)–Br	−8.10	−4.64	3.46	1.73	0.29	1.25
**C4**	Ni(II)–Cl	−8.08	−6.06	2.02	1.01	0.50	2.63

A critical mechanistic insight emerges from comparing the isostructural cobalt complexes. The axial replacement of chloride (**C1**) by bromide (**C3**) reduced the energy gap from 3.72 to 3.46 eV and increased chemical softness from 0.27 to 0.29 eV^−1^. This increased softness indicates enhanced electron‐cloud polarizability in **C3**, thereby fostering a more lipophilic profile. Pharmacokinetically, this enhanced lipophilicity may favor passive diffusion across the lipid bilayers of the infected erythrocyte and the parasite. This theoretical rationale aligns with the in vitro data, in which **C3** achieved the highest biological potency (IC_50_ = 1.25 µM against the 3D7 strain), demonstrating that its superior ability to reach effective intracellular concentrations is intrinsically linked to its tuned electronic structure.

#### 3.5.2 Theoretical Estimation of Lipophilicity

The theoretically calculated partition coefficients reveal a pronounced lipophilic character across all four coordination complexes, as summarized in Table 6. These highly positive log *P* values (ranging from 13.30 to 14.43) are a direct consequence of the massive hydrophobic shield provided by the peripheral *tert*‐butoxy groups surrounding the coordination sphere.

**TABLE 6:** Thermodynamic solvation energies and calculated partition coefficients (log *P*) at the SMD/M06‐2X/6‐31+G(d, p)/LANL2DZ level of theory.

Complex	Δ*G*_solv_ Octanol, kcal/mol	Δ*G*_solv_ Water, kcal/mol	ΔΔ*G*_tr_, kcal/mol	log*P*
**C1 (Co–Cl)**	−62.34	−43.90	−18.44	13.52
**C2 (Cu–Cl)**	−62.41	−42.73	−19.68	14.43
**C3 (Co–Br)**	−62.16	−44.02	−18.14	13.30
**C4 (Ni–Cl)**	−61.65	−42.86	−18.79	13.77

While complex **C2** (Cu–Cl) exhibited the highest absolute lipophilicity (log *P* = 14.43), the most biologically active complex, **C3** (Co–Br), presented a slightly lower and potentially more balanced lipophilic profile (log *P* = 13.30). In the context of bulky metallodrugs, extreme lipophilicity can frequently lead to sequestration of the compound within the host erythrocyte membrane, preventing the drug from reaching the intracellular parasite. Therefore, the modulated partition coefficient of **C3**, synergistically combined with its enhanced chemical polarizability, provides an optimal pharmacokinetic equilibrium. It is lipophilic enough to effortlessly traverse the parasitic lipid bilayers via passive diffusion yet sufficiently balanced to avoid permanent membrane entrapment.

#### 3.5.3 Molecular Docking and Binding Mode Analysis: PfLDH Target

Evaluating the classical absorption, distribution, metabolism, excretion, and toxicity (ADMET) properties of coordination compounds in silico remains inherently challenging. Standard predictive algorithms are primarily parameterized for organic molecules and often fail to adequately describe the coordination environment and relativistic effects associated with transition metals. Consequently, rather than attempting to predict systemic ADMET profiles, quantum global reactivity descriptors derived from FMO theory were employed as fundamental physicochemical indicators to assess the intrinsic reactivity and kinetic stability of complexes **C1**–**C4**. While these electronic parameters cannot replace comprehensive empirical pharmacokinetic data, when combined with thermodynamic solvation models, they provide a robust molecular‐level rationale for estimating passive membrane permeability and the capacity of the complexes to reach intracellular targets.

To elucidate the atomistic details of the antiplasmodial activity, molecular docking simulations were performed against *P. falciparum* Lactate Dehydrogenase (PfLDH, PDB ID: 1T24). To establish a thermodynamic baseline and validate the docking protocol, the cocrystallized azole‐based inhibitor was redocked, yielding a reference binding affinity of −9.0 kcal/mol within the catalytic site. The coordination complexes exhibited highly favorable binding affinities and theoretical inhibition constants (K_i_), as summarized in Table 7.

**TABLE 7:** Binding affinities and theoretical inhibition constants (K_i_) for complexes C1–C4 against PfLDH (PDB ID: 1T24).

Complex	Metal–Halogen core	Affinity, Δ*G*, kcal/mol	K_i_, μM
**C1**	Co–Cl	−7.81	1.87
**C2**	Cu–Cl	−7.90	1.63
**C3**	Co–Br	−7.64	2.50
**C4**	Ni–Cl	−7.99	1.40
**Ref.**	NAD	−9.00	0.25

**FIGURE 9:**
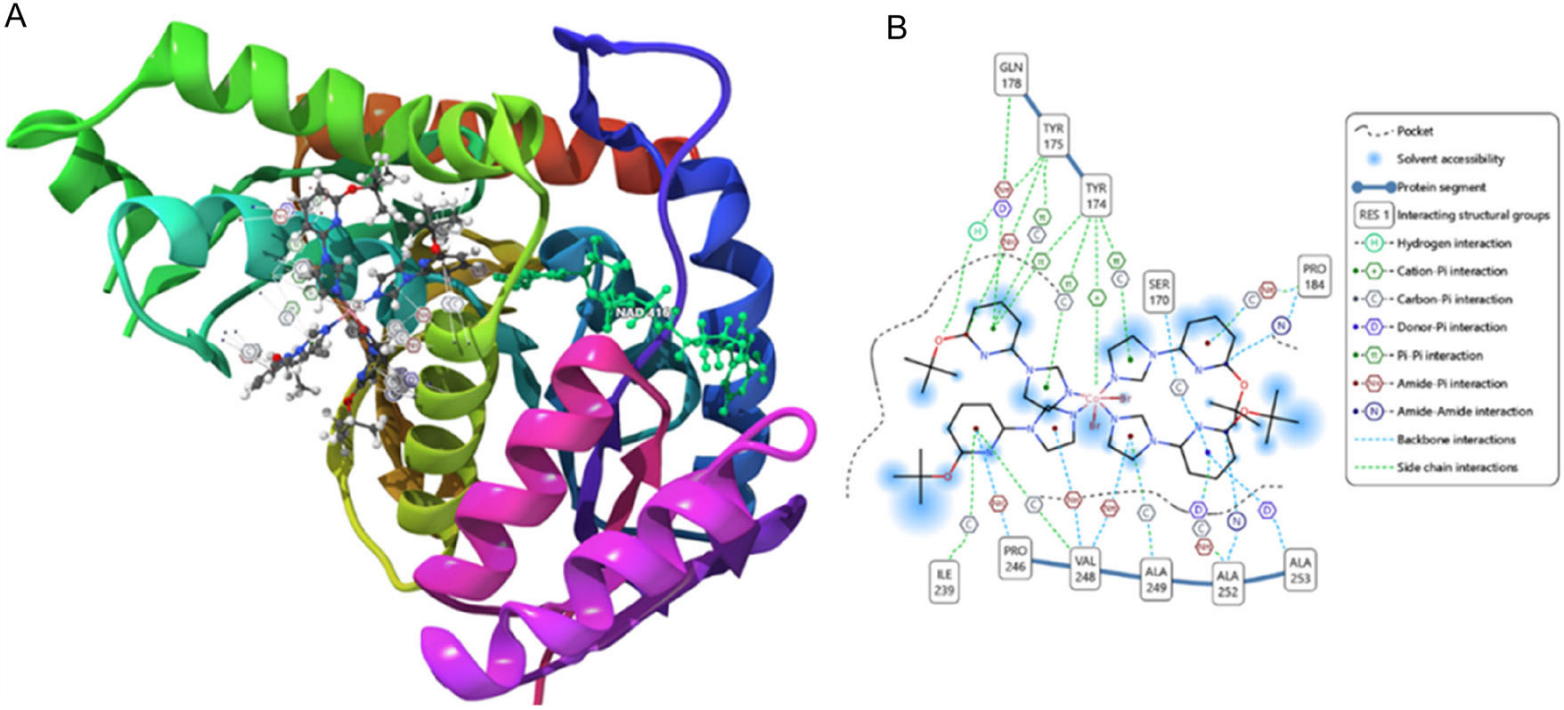
(A) Structural superposition of complex **C3** and NAD (green) within the *Pf*LDH framework, highlighting the occupancy of the secondary allosteric pocket. (B) Detailed 2D ligand‐interaction diagram for **C3** showing π‐stacking and hydrophobic anchoring with key residues.

Crucially, structural superposition and visual inspection of the optimal binding poses, particularly for the active complex **C3** (Co–Br), revealed a striking binding paradigm. As illustrated in Figure 9A, while the native NAD cofactor occupies the highly conserved catalytic cleft, the massive steric volume of the octahedral complexes appears to prevent deep penetration into this primary cavity. Instead, the docking model predicts that **C3** favorably anchors into a distinct, secondary pocket separate from the NAD(H) domain.

This spatial divergence suggests a putative noncompetitive or allosteric‐like mechanism of inhibition. The **L** ligands established a robust network of noncovalent interactions within this alternative pocket. Dominant π‐interactions characterize the binding mode, including strong π–π stacking with Tyr174 and Tyr175, further stabilized by cation‐π and amide‐π interactions with the backbone and side chains of Gln178, Pro246, and Ala252.

Additionally, the complex is anchored by a dense network of Van der Waals forces and backbone interactions with Ala249, Ala252, Ala253, and Val248 (Figure 9B), while the hydrophobic side chain of Ile239 stabilizes the peripheral aromatic rings. Based on this architectural binding model, we hypothesize that the antiplasmodial efficacy of these compounds could arise from a dual‐action interference: providing a steric hindrance that may restrict the transition of the specificity loop and imposing conformational constraints upon the enzyme. While further kinetic and molecular dynamics studies are required to confirm this allosteric behavior, targeting this secondary binding site provides a compelling structural rationale for the observed selectivity against the parasite enzyme, potentially circumventing the highly conserved cofactor pockets found in human LDH isoforms.

#### 3.5.4 Molecular Docking and Binding Mode Analysis: Falcipain‐2 (PfFP‐2) Target

To further explore the multitarget potential of the coordination complexes, docking simulations were performed against Falcipain‐2, a major cysteine protease strictly involved in the degradation of host hemoglobin within the parasite's digestive vacuole. This catabolic process is vital for providing essential amino acids required for intraerythrocytic parasite survival and development [97]. To validate the docking protocol, the reference cysteine protease inhibitor E‐64 was docked into the active site, yielding a binding affinity of −7.98 kcal/mol. The coordination complexes exhibited high consistency and competitive affinities, with values ranging from −7.04 to −7.50 kcal/mol (Table 8).

**TABLE 8:** Binding affinities (Delta G) and theoretical inhibition constants (K_i_) for complexes **C1–C4** against Falcipain‐2 (PfFP‐2).

Complex	Metal–Halogen core	Affinity, Δ*G*, kcal/mol	K_i_, μM
**C1**	Co–Cl	−7.05	6.84
**C2**	Cu–Cl	−7.04	6.95
**C3**	Co–Br	−7.08	6.58
**C4**	Ni–Cl	−7.50	3.17
**Ref.**	E‐64	−7.98	1.41

**FIGURE 10:**
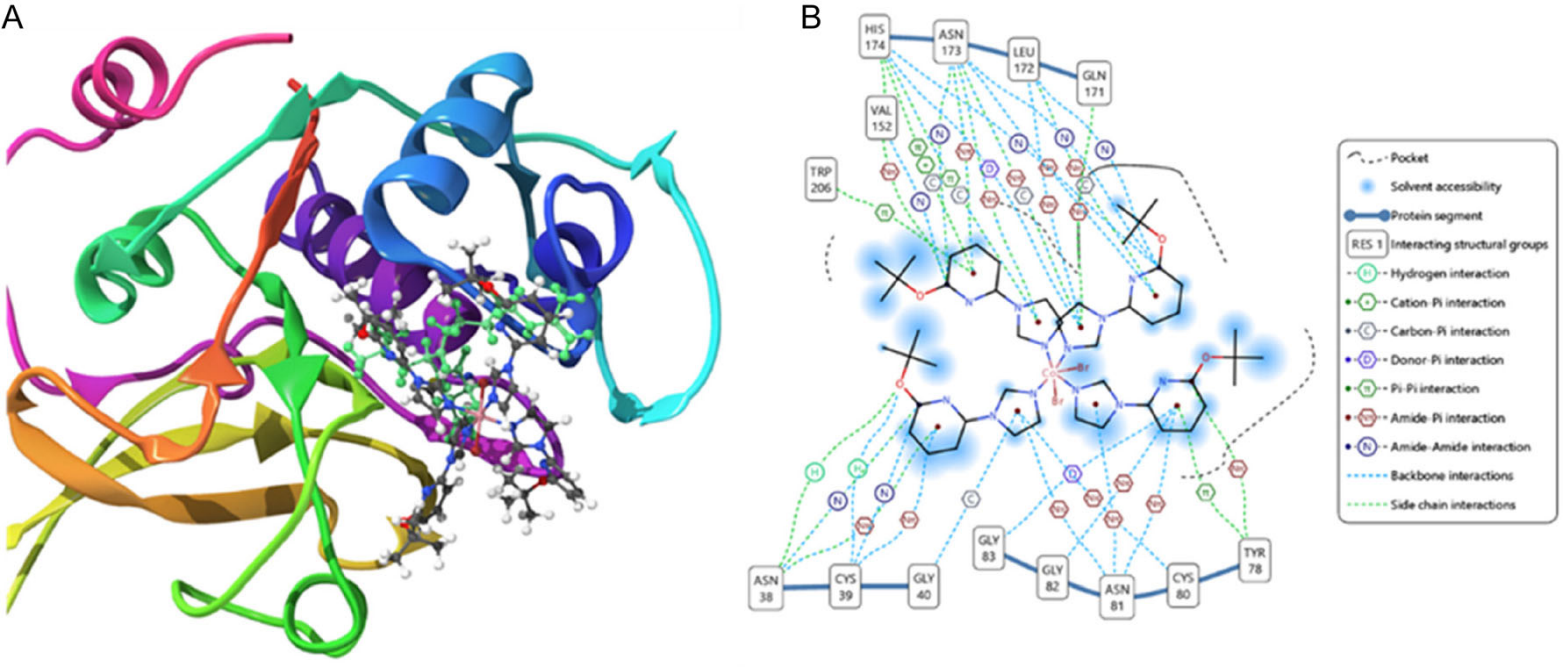
(A) Structural 3D superposition of complex **C3** (represented in CPK colors) and the reference inhibitor within the Falcipain‐2 catalytic site in green. (B) Detailed 2D ligand‐interaction diagram for **C3** highlighting the network of π‐stacking, hydrogen bonding with Asn38/Cys39, and the steric fit of the peripheral *tert*‐butoxy groups.

Structural analysis through superposition (Figure 10A) indicates that the octahedral complexes can anchor within the catalytic cleft, potentially blocking the groove and restricting substrate access. Complex **C3** (Co–Br) exhibits a computationally predicted binding mode characterized by a putative network of hydrogen bonds and π‐interactions. As illustrated in the 2D interaction diagram (Figure 10B), the aromatic scaffold appears to be stabilized by π–π stacking with Trp205 and Tyr78, while the imidazole rings are positioned to engage in multiple amide‐π and cation‐π interactions with residues including Asn173, His174, and Gln171.

Furthermore, the model suggests that the complex is stabilized by backbone and side‐chain interactions with the sulfur‐rich region near the catalytic dyad, involving Cys39, Cys80, and Gly82. The bulky *tert*‐butoxy groups are predicted to provide steric complementarity, potentially anchoring the complex against Asn38 and Val152. This interaction pattern supports the hypothesis that the antiplasmodial activity of these compounds may be partially mediated by the physical obstruction of the catalytic cleft, directly impairing hemoglobin proteolysis and subsequently disrupting the parasite's nutrient supply.

While the molecular docking simulations provide valuable structural insights into potential binding paradigms, a direct linear correlation between the in silico thermodynamic binding affinities and the in vitro biological potencies is not strictly observed within this series. Notably, although complex **C4** (Ni–Cl) exhibits the most favorable docking binding energies across both predicted targets (*Pf*LDH and *Pf*FP‐2), complex **C3** (Co–Br) demonstrates the highest antiplasmodial potency in vitro (IC_50_ = 1.25 µM).

This apparent discrepancy must be explicitly addressed and highlights the multifactorial nature of whole‐cell phenotypic assays, where isolated receptor affinity is only one component of overall efficacy. The superior biological activity of **C3** is likely governed by pharmacokinetic and membrane permeability factors rather than pure pharmacodynamics. As indicated by the DFT and thermodynamic solvation models, the Co–Br core in **C3** confers a unique electronic signature characterized by an increased chemical softness (0.29 eV^−1^) and a slightly more modulated lipophilicity (log *P* = 13.30) compared to the Ni–Cl core in **C4** (softness = 0.50 eV^−1^, log *P* = 13.77).

In the context of the intraerythrocytic *Plasmodium* lifecycle, an antimalarial drug must cross multiple lipid barriers, the host RBC membrane, the parasitophorous vacuole membrane, and the parasite plasma membrane before reaching its intracellular targets. The finely tuned lipophilic and polarizable profile of **C3** may facilitate superior passive diffusion across these extensive lipid bilayers, minimizing membrane entrapment and leading to a higher effective intracellular accumulation. Consequently, although **C4** may form a slightly more stable complex once positioned within the active sites of the isolated enzymes, the enhanced bioavailability and membrane permeation kinetics of **C3** ultimately drive its superior phenotypic antiplasmodial activity.

## 4 Conclusions

Four coordination complexes (**C1–** **C4**) with octahedral geometry were obtained from the ligand 2‐(tert‐butoxy)‐6‐(1H‐imidazol‐1‐yl)pyridine and Cu(II), Co(II), Ni(II) metal centers, which were purified as crystalline solids in good yields and with stability in air and solution. The structure comprises four ligands and two coligand halogens, as corroborated by various analytical, spectroscopic, and X‐ray techniques. Thermal stability analysis showed that the complexes exhibit high thermal resistance. Furthermore, conductivity studies in acetonitrile suggest a nonelectrolyte behavior. These results indicate that the coligand anions remain largely coordinated to the metal center. In addition, the intrinsic fluorescence of the free ligand, with a weak emission at 390 nm, is completely quenched upon coordination with the metal. Topological analysis using ToposPro confirmed that all four complexes share the same supramolecular network topology (1,6M7−1). The identification of two nonequivalent nodes (ZA1 and ZA2) for the same ligand reflects distinct supramolecular interactions, consistent with local distortions around each metal center. This topology, not previously reported in the RCSR database, defines a new structural archetype for these systems. The four metal complexes showed antiplasmodial activity against both chloroquine‐sensitive and resistant *P. falciparum* strains. While potency varied among compounds, integrating antiplasmodial activity, hemolytic profile, selectivity analysis, and computational physicochemical descriptors identified **C3** and **C4** as the most promising candidates, displaying an optimal balance between efficacy and safety. Computational analyses further suggested that modulation of the electronic and lipophilic properties induced by metal coordination may contribute to the distinct biological profiles observed among the evaluated complexes. In particular, the comparatively balanced lipophilic and polarizable profile predicted for C3 may favor membrane interactions and intracellular accessibility across the multiple lipid barriers encountered during the intraerythrocytic parasite lifecycle, potentially contributing to its superior phenotypic antiplasmodial potency.

The comparable activity of **C4** across parasite phenotypes further suggests a more consistent inhibitory profile across strains, whereas the moderate effect of the free ligand highlights the contribution of metal coordination to biological performance. Additionally, molecular docking analyses against PfLDH and PfFP‐2 supported the possibility of multitarget interactions involving steric and hydrophobic contacts within parasite‐associated enzymatic regions, providing a plausible structural rationale for the observed antiplasmodial behavior. Collectively, these results support the potential of the evaluated metal‐based complexes as promising candidates for antimalarial development and warrant further mechanistic and cytotoxicity studies.

## Author Contributions

D.E.‐S, D.F.‐L. and A.P.‐C carried out the synthesis and characterization of the ligand and complexes. M.A.M. and C.S.‐S. performed the crystallographic and solid‐state analysis. M.C.V.‐P., A.R., and M.F.Y.‐A. carried out the biological assays. All authors wrote the original draft and contributed with crucial discussions and constructive reviews.

## Funding

This study was supported by Facultad de Ciencias, Universidad de los Andes (2023‐176‐2938, 2025–213‐3347), Universidad de los Andes (PVI0122029).

## Ethics Statement

This article does not contain any studies with human participants performed by any of the authors.

## Conflicts of Interest

The authors declare no conflicts of interest.

## Supporting information

Supplementary Material

## Data Availability

No datasets were generated or analyzed during the current study.
