# Supplementary material for: Co(II), Cu(II), and Ni(II) Coordination Complexes: Synthesis, Characterization, Experimental, and Computational Study on Potential Antiplasmodial Activity
Source: ChemMedChem. 2026 Jun 22;21(12):e70347. doi: 10.1002/cmdc.70347 (PMC13286813; doi:10.1002/cmdc.70347)
Supplement: Supplementary file 1 — Supplementary Material [file CMDC-21-e70347-s001.pdf]

Co(II), Cu(II) and Ni(II) Coordination Complexes: Synthesis, Characterization, experimental and computational study on potential antiplasmodial

David Ezenarro-Salcedo [a], Daniela Fonseca-López [a], Alexander Patiño-Cubides [a], María C. Velasco-Pareja [b], María F. Yasnot-Acosta [b], Camilo Serrano-Sterling [c], Ana Rodríguez [d], Mario A. Macías [c], Augusto Valderrama-Aguirre [e], Adrian L. Orjuela [f] and John J. Hurtado [a]*

Content

1. Material and methods 1

2. Characterization of L 2

3. Characterization of Metal complexes 4

4. Crystallographic data. 21

5. Biological activity 22

1. Material and methods

All metal precursors were used as received from the respective companies ($CoCl_2 \cdot 6H_2O$ (purity, 98%), $CuCl_2 \cdot 2H_2O$ (purity, 99%), $CoBr_2$ (Purity, 98%), Merk, $NiCl_2$ (Purity, 98%), Alfa Aesar). Elemental analysis (C, H, and N) was performed with a Thermo Scientific™ FLASH 2000 CHNS/O Analyzer. Fourier transform infrared (FTIR) spectra were recorded on a Shimadzu IR Tracer-100 spectrophotometer (Shimadzu Corporation, Kyoto, Japan) by ATR in the range of 4000 to 400 cm^{-1}. Melting points were determined on a Mel-Temp® 1101D apparatus in open capillary tubes and are uncorrected. The electronic UV/Vis absorption spectra were measured from 200 to 800 nm in DCM solution in a quartz cuvette with a 1cm optical path length using a Varian Cary 100 spectrophotometer (Agilent Technologies, Santa Clara, CA, USA). Raman spectroscopy was performed on an XploRA-HORIBA Scientific spectrometer using a 532 nm laser. Thermogravimetric (TG) analyses of the complexes were conducted on a DISCOVERY TGA550 using 8-10 mg samples in a nitrogen atmosphere. Samples were subjected to dynamic heating over a temperature range of 30-950 °C at a heating rate of 10 °C min^{-1}. TG curves were analyzed to obtain the percent mass losses as a function of temperature. Nuclear magnetic resonance (NMR) spectra were recorded at **L** on a Bruker Ascend™-400 spectrometer at 295 K. Chemical shifts are reported in ppm relative to $SiMe_4$ (^1H) as an internal standard. ^1H-NMR chemical shifts (δ) are reported in parts per million (ppm) relative to TMS, with the residual solvent peak used as an internal reference; $CDCl_3$ (^1H-NMR d: 7.26) Molar conductivity measures ($\Lambda_M \equiv \Omega^{-1}cm^2mol^{-1}$) were obtained in an OAKTON WD-35413-21 pH/CON 700 equipment, using acetonitrile as a solvent, with a

concentration of 1.0 mM for the complexes, to 19 °C. Fluorescence measurements were performed on a Cary Eclipse spectrophotometer (Agilent Technologies, Santa Clara, CA, USA) using pure samples of the ligands and the corresponding coordination complexes.

The X-ray diffraction data were acquired at room temperature using Cu Kα (λ = 1.54184 Å) radiation. For the measurements, an Agilent SuperNova Dual Cu at Zero Atlas four-circle diffractometer equipped with a CCD plate detector was used for w scans. The collected frames were integrated and corrected for the absorption effect using the CrysAlis PRO software package (CrysAlisPro 1.171.39.46e, Rigaku Oxford Diffraction, 2018). The crystal structure was solved using an iterative algorithm [29] and completed by a difference Fourier map. The crystal structures were refined using the program SHELXL2018/3 [30]. Molecular and supramolecular graphics were generated using the Mercury software [31]. The Crystallographic Information File (CIF) files have been deposited with the Cambridge Crystallographic Data Center (CCDC) under deposition numbers CCDC-2463438, 2463436, 2463439, and 2463437 for compounds **C1**, **C2**, **C3**, and **C4**, respectively.

2. Characterization of L

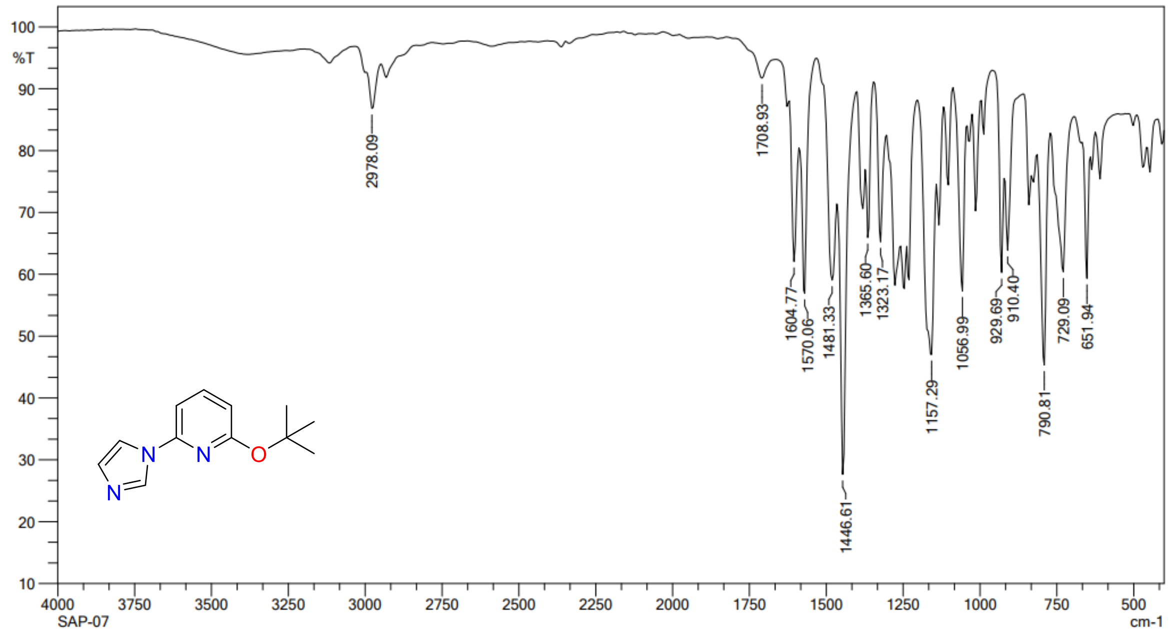

Figure S1. FT-IR spectrum of **L**.

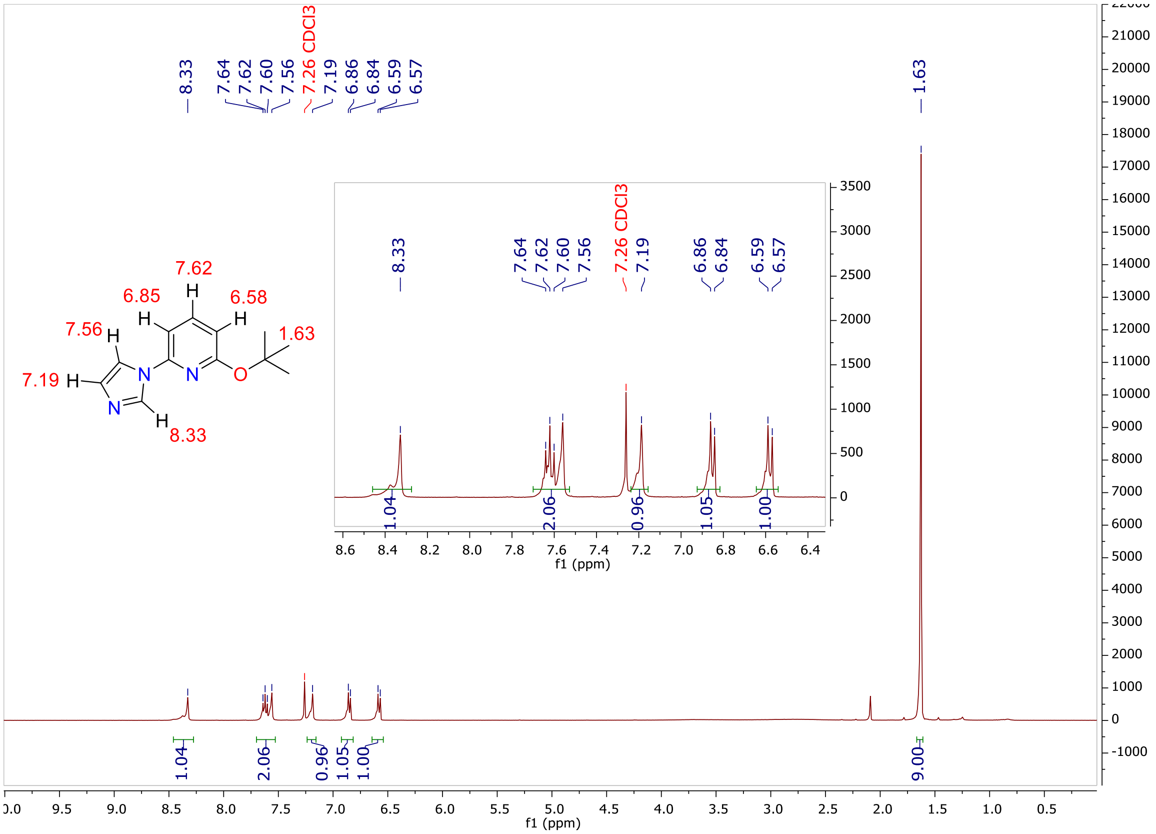

Figure S2. ^{1}H-NMR spectrum of **L** in $CDCl_3$.

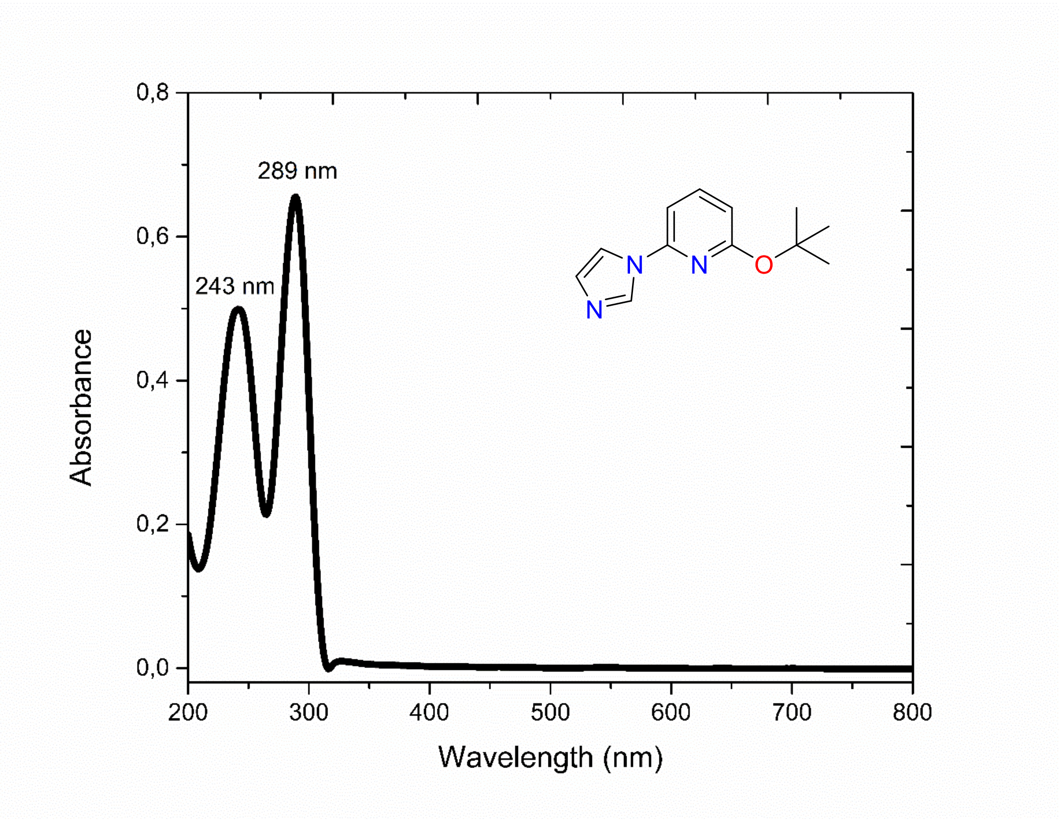

Figure S3. UV-Vis spectrum of **L** in DCM (5 x 10^{-6} M).

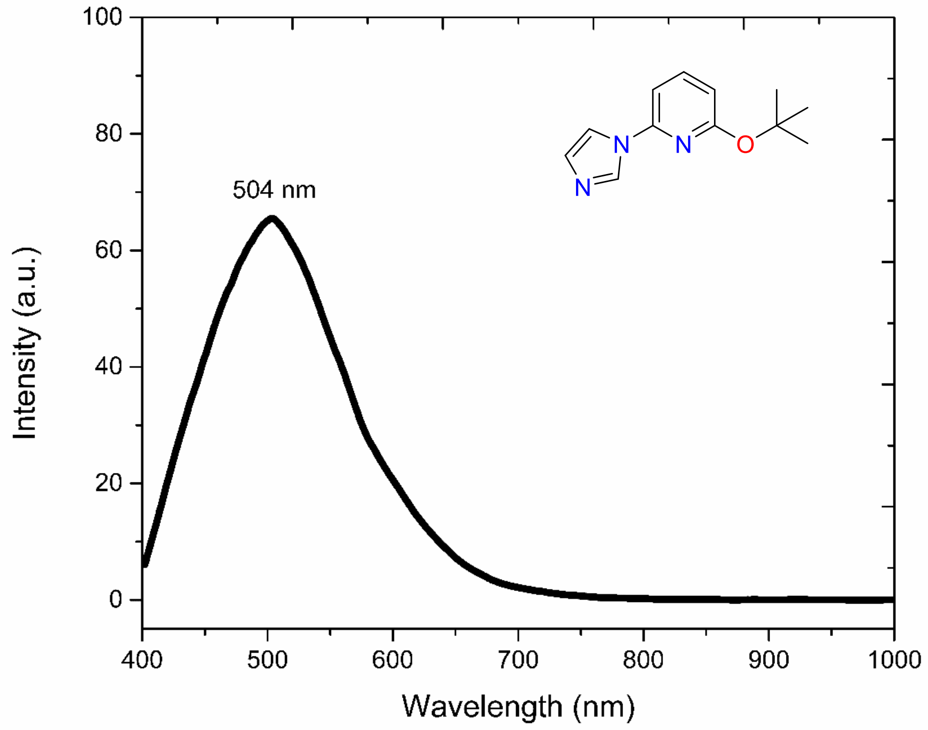

Figure S4. Emission spectrum of **L** (λ_{ex}: 390 nm).

3. Characterization of Metal complexes

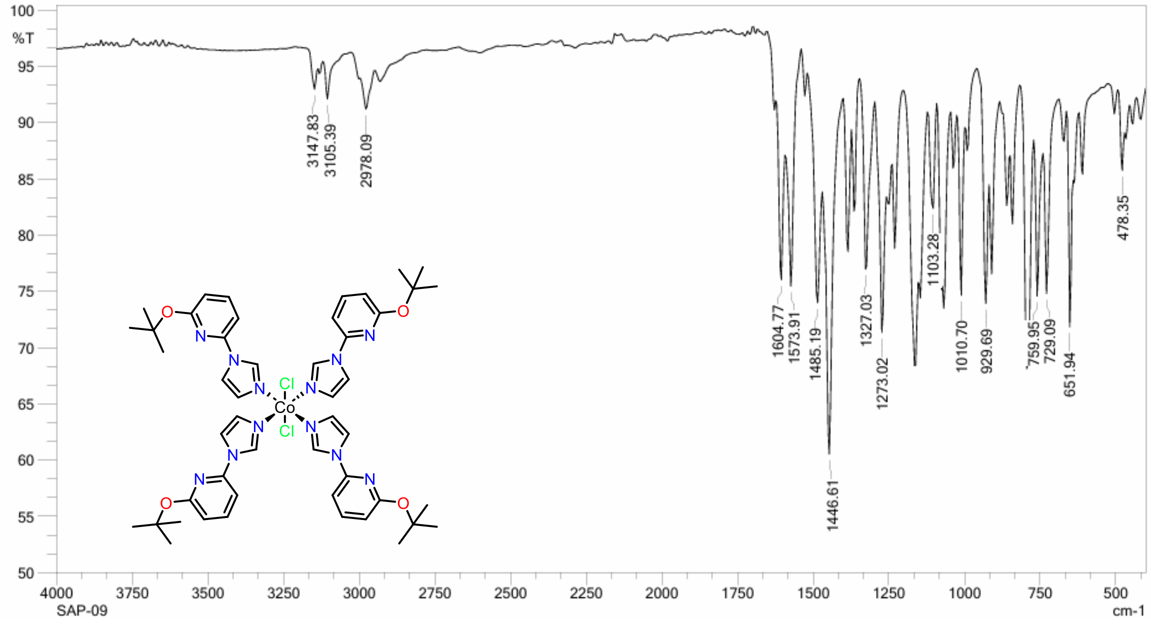

Figure S5. FT-IR spectrum of **C1**.

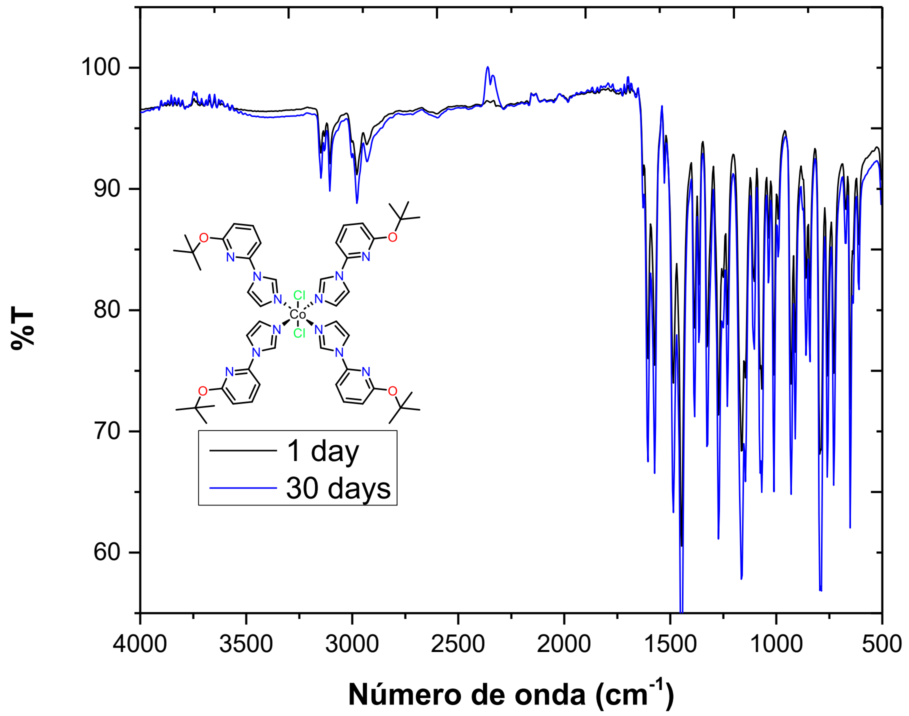

Figure S6. FT-IR spectrum of **C1** for air-stability.

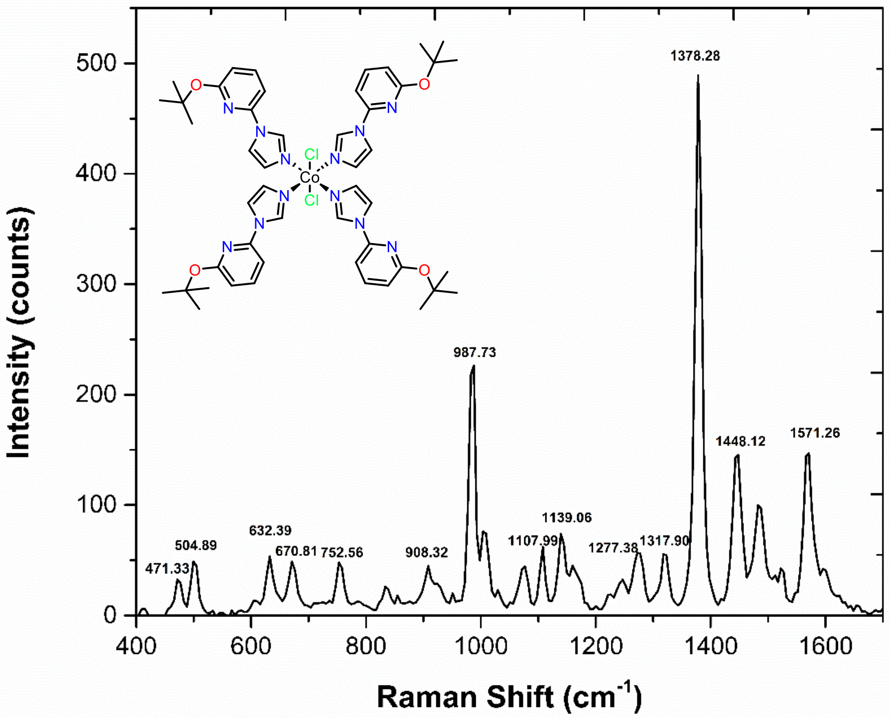

Figure S7. Raman spectrum of **C1.**

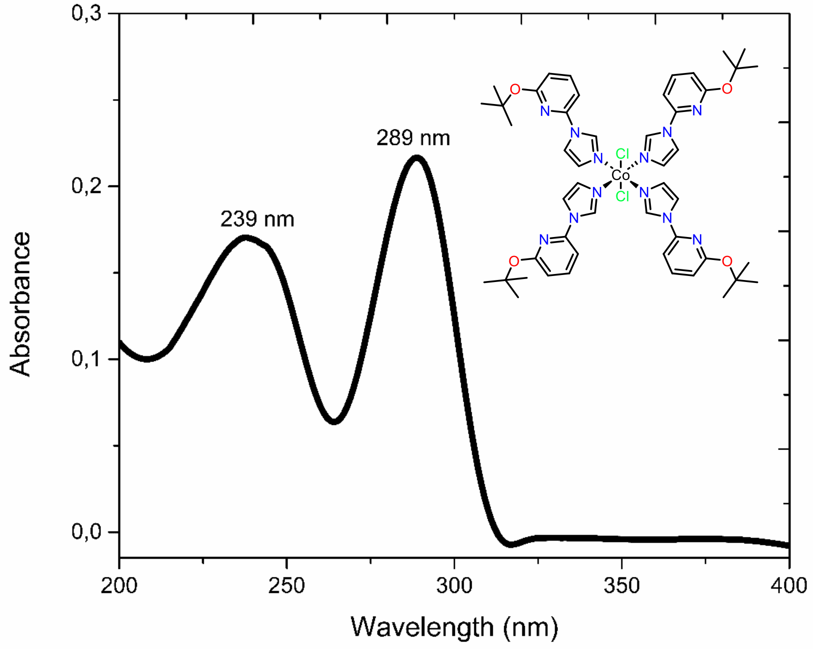

Figure S8. UV Absorption spectrum of **C1** in DCM (5 x 10^{-6} M)**.**

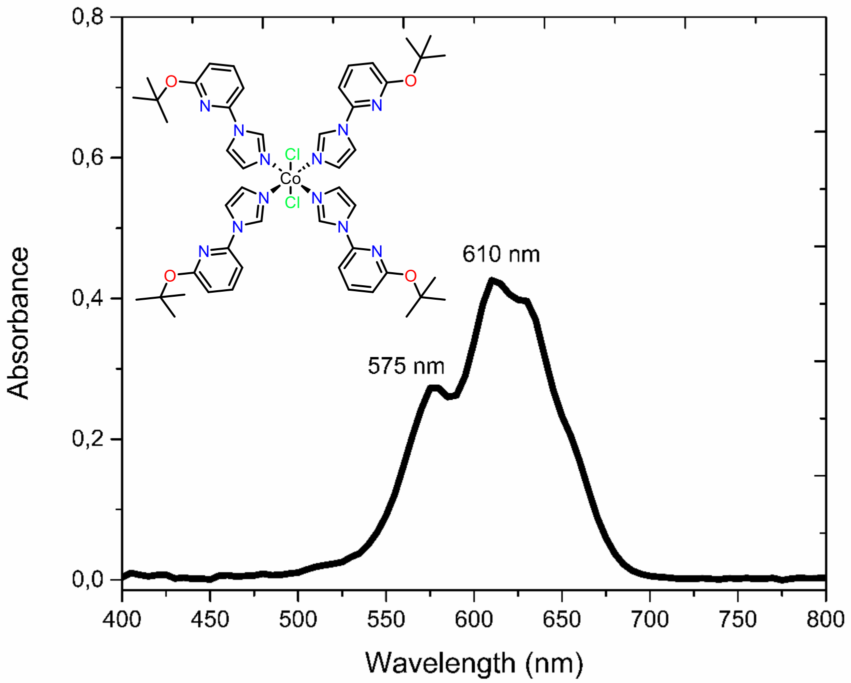

Figure S9. Visible Absorption spectrum of **C1** in DCM (5 x 10^{-4} M).

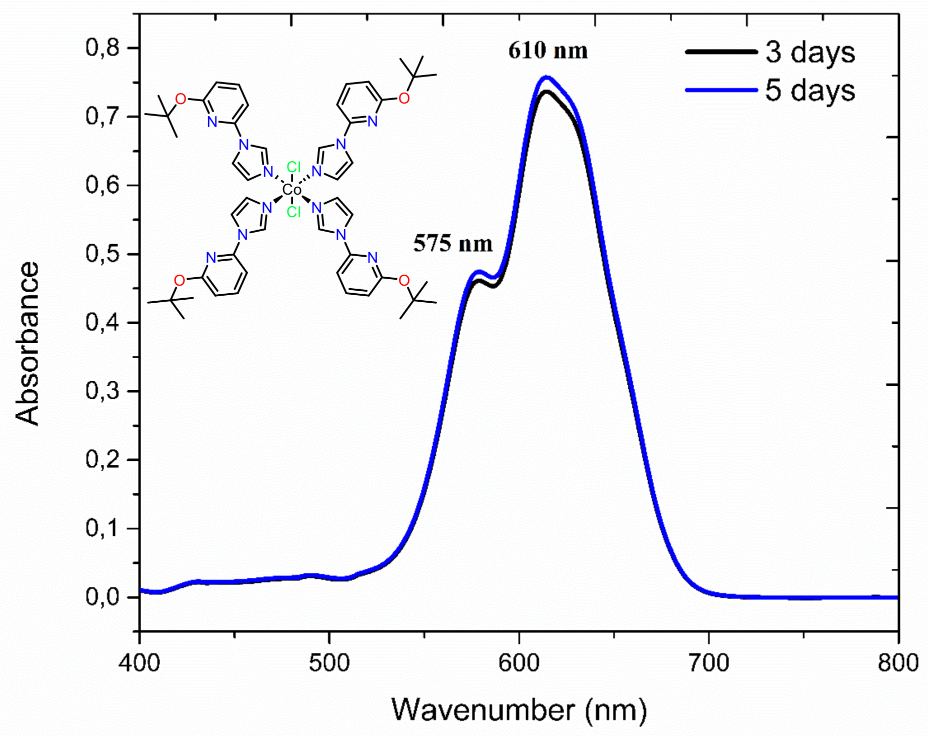

Figure S10. Visible Absorption spectrum of **C1** in DCM (1 x 10^{-3} M) for stability in solution.

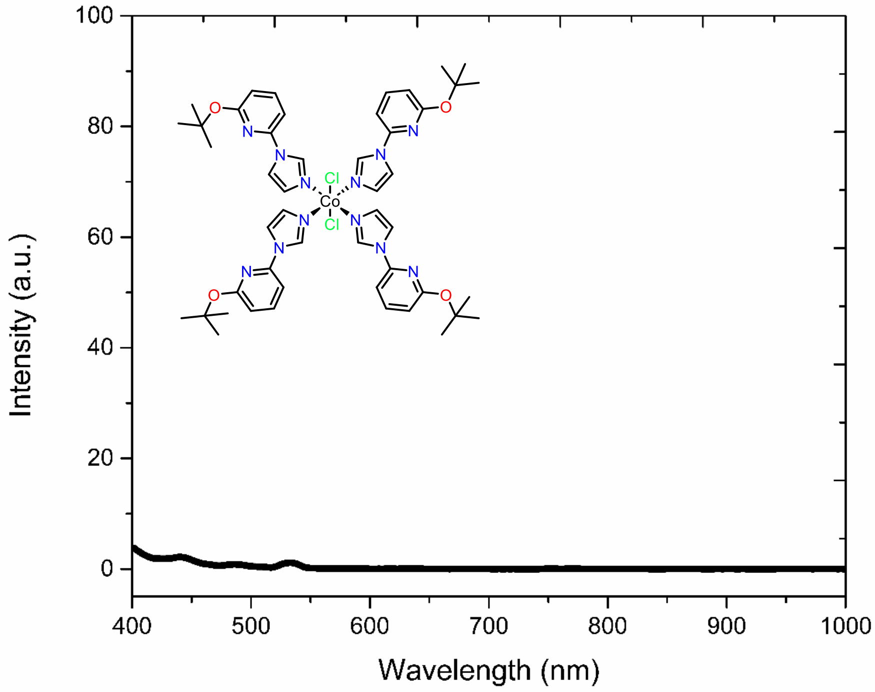

Figure S11. Emission spectrum of **C1** (λ_{em}: 390 nm).

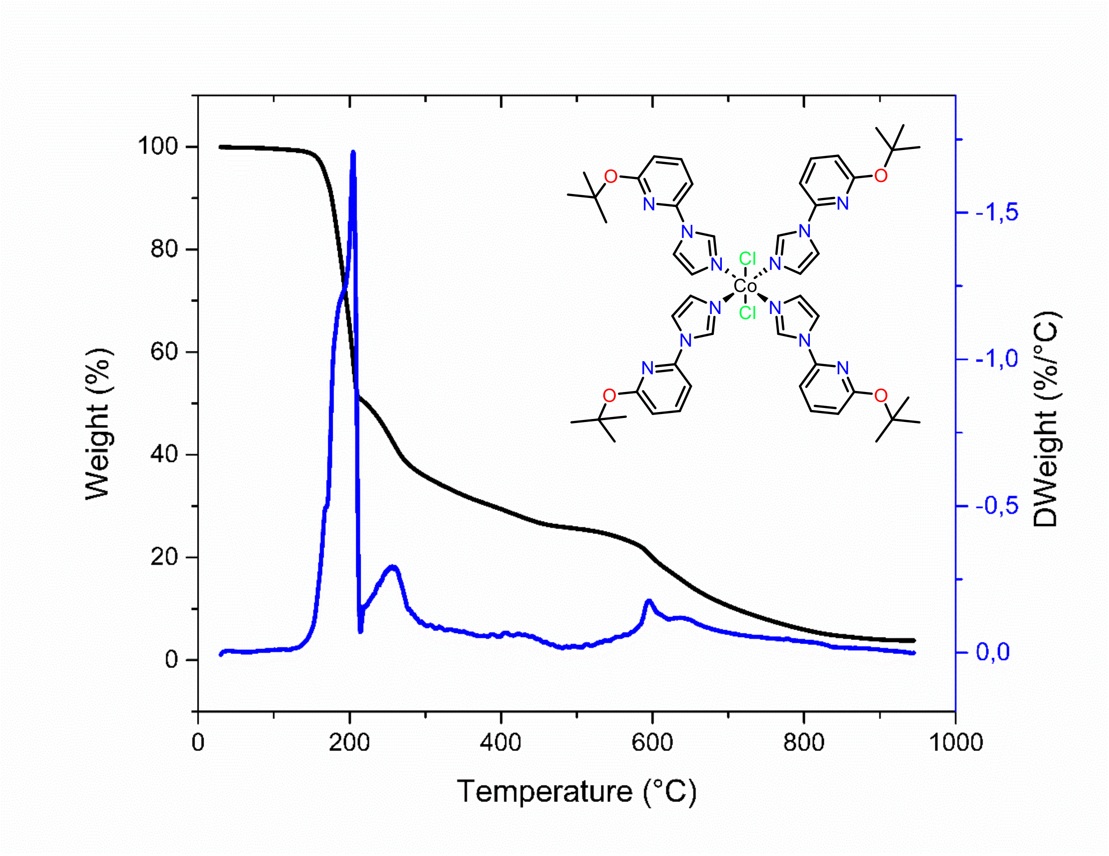

Figure S12. Thermogram of **C1**.

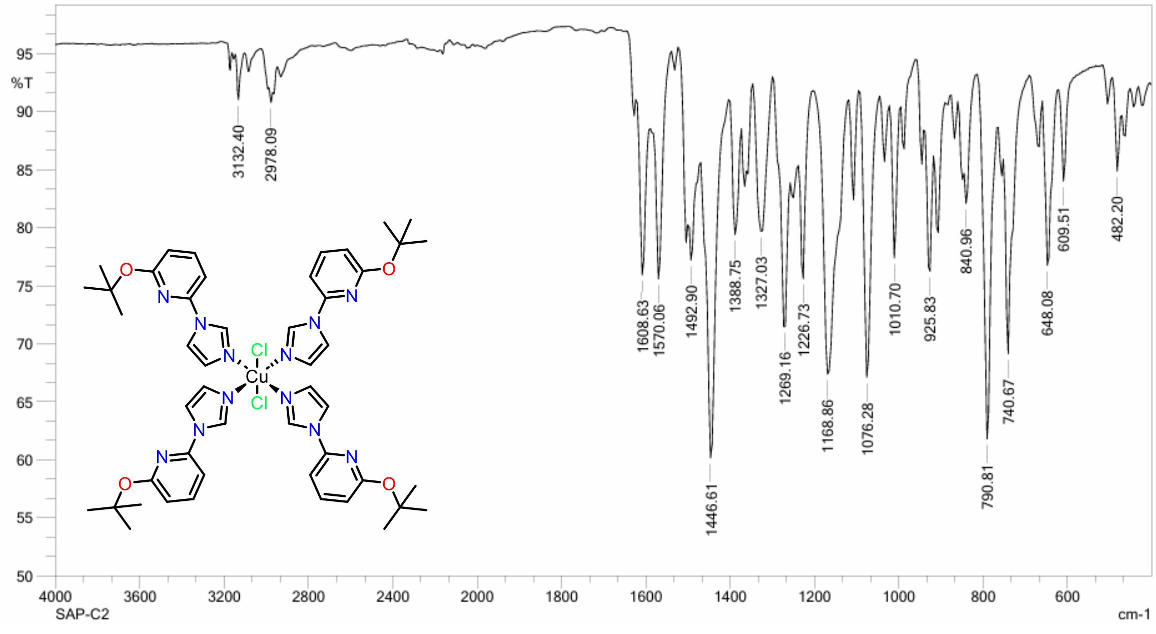

Figure S13. FT-IR spectrum of **C2**.

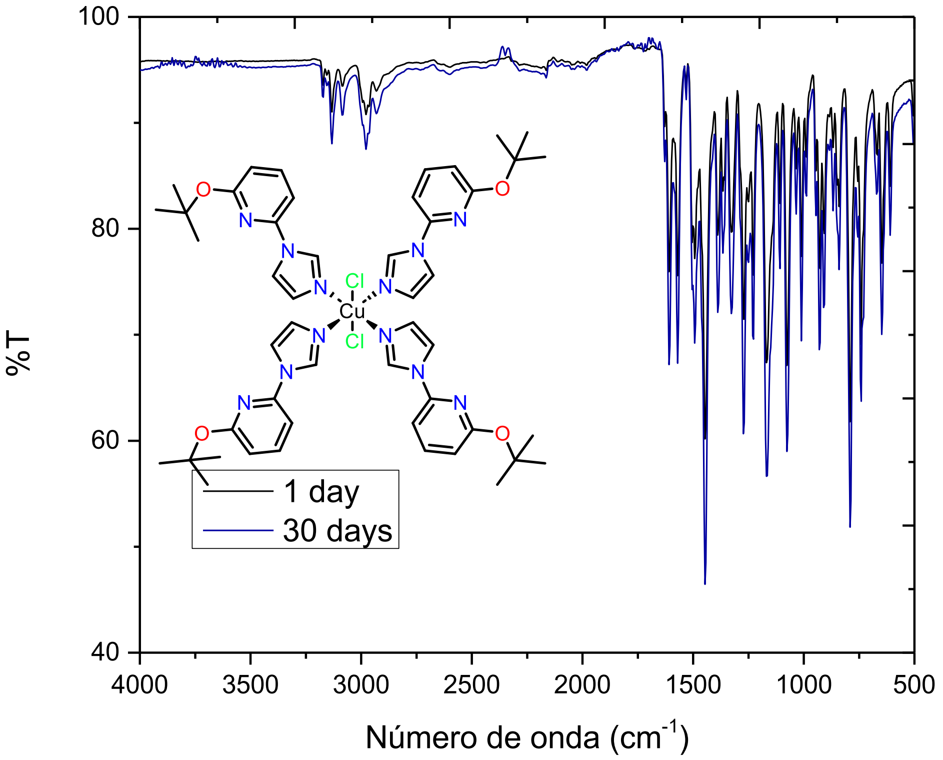

Figure S14. FT-IR spectrum of **C2** for air stability

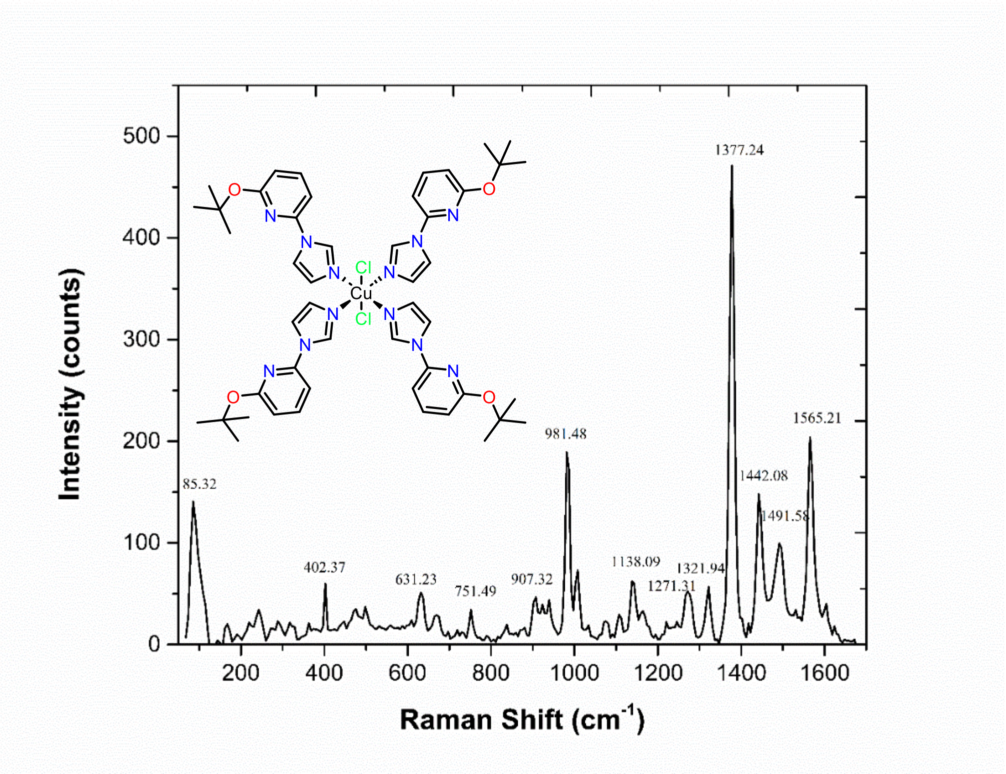

Figure S15. Raman spectrum of **C2**.

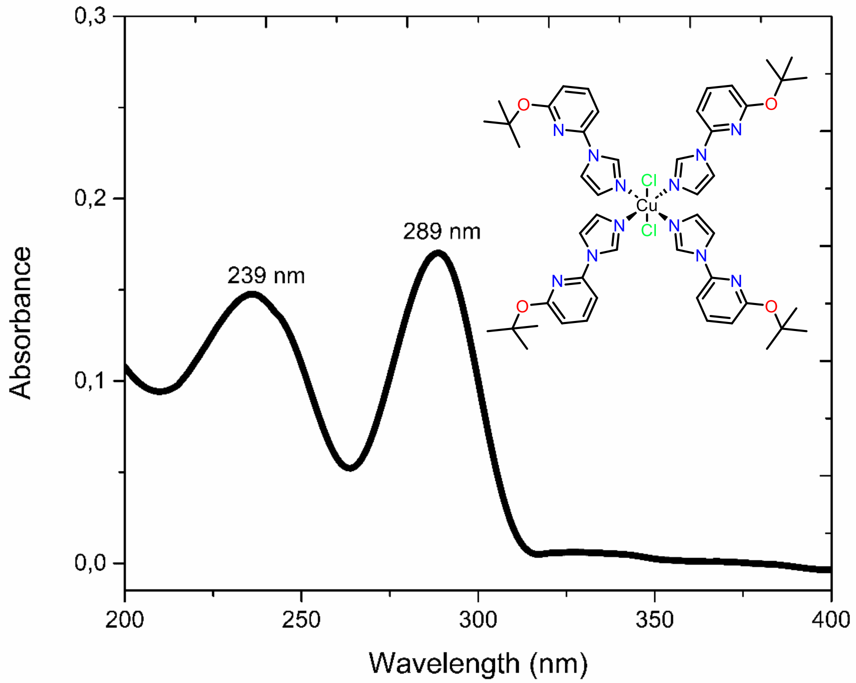

Figure S16. UV Absorption spectrum of **C2** in DCM (5 x 10^{-6} M).

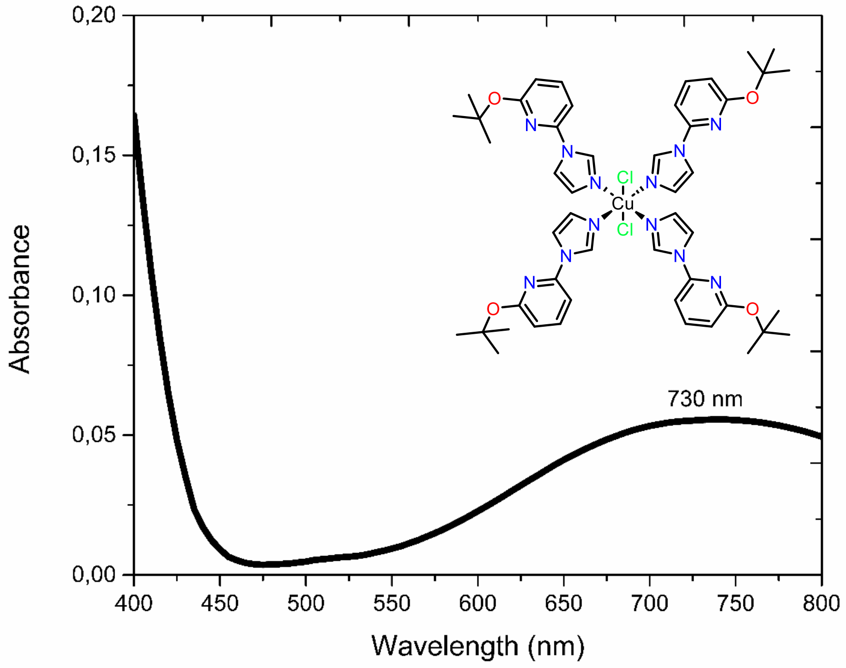

Figure S17. Visible Absorption spectrum of **C2** in DCM (5 x 10^{-4} M).

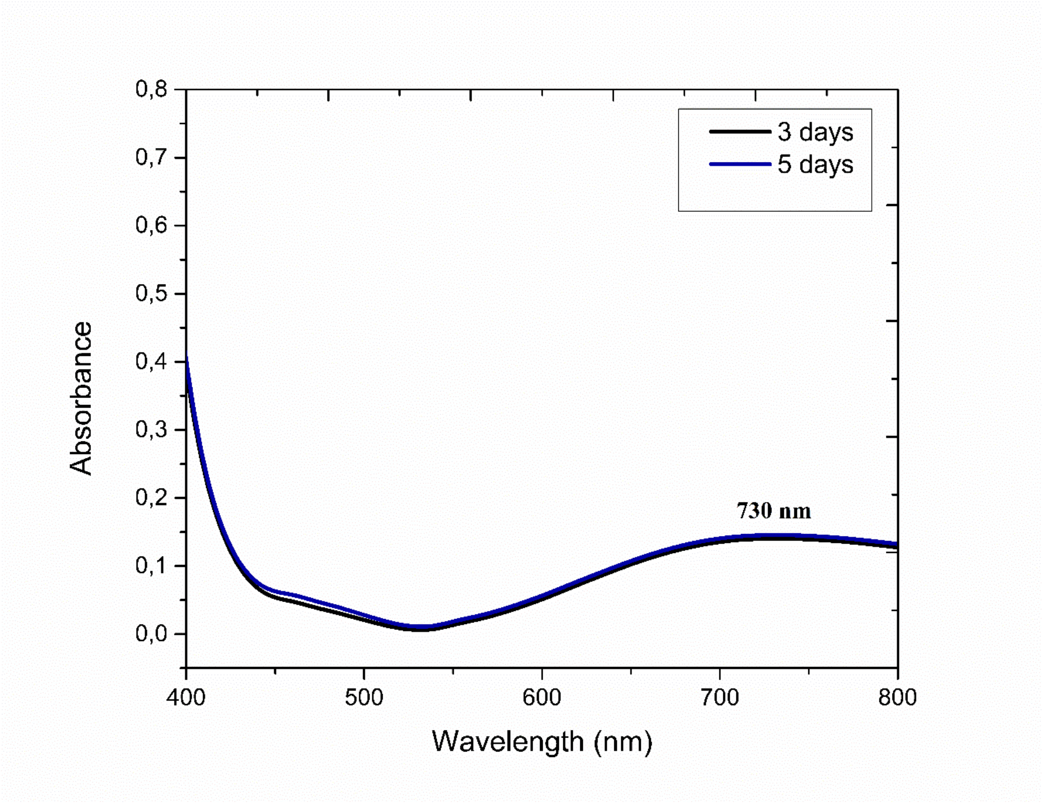

Figure S18. Visible Absorption spectrum of **C2** in DCM (1 x 10^{-3} M) for stability in solution.

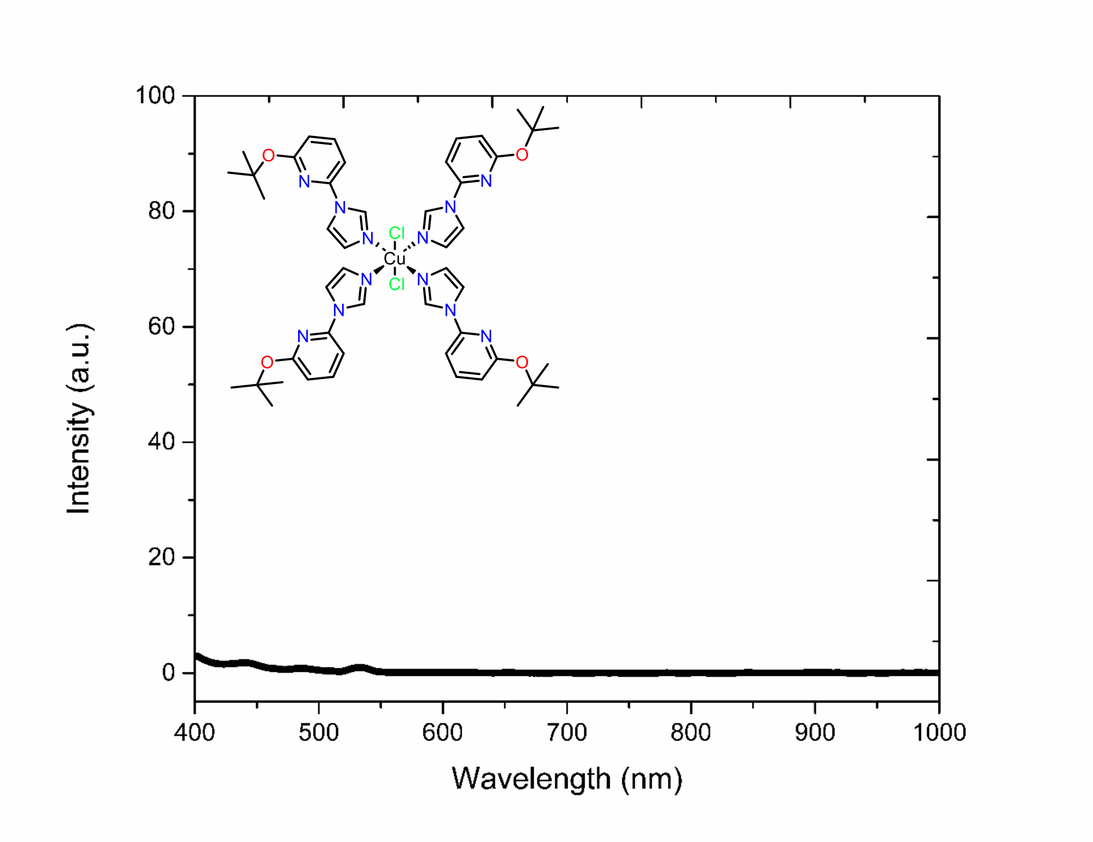

Figure S19. Emission spectrum of **C2** (λ_{em}: 390 nm).

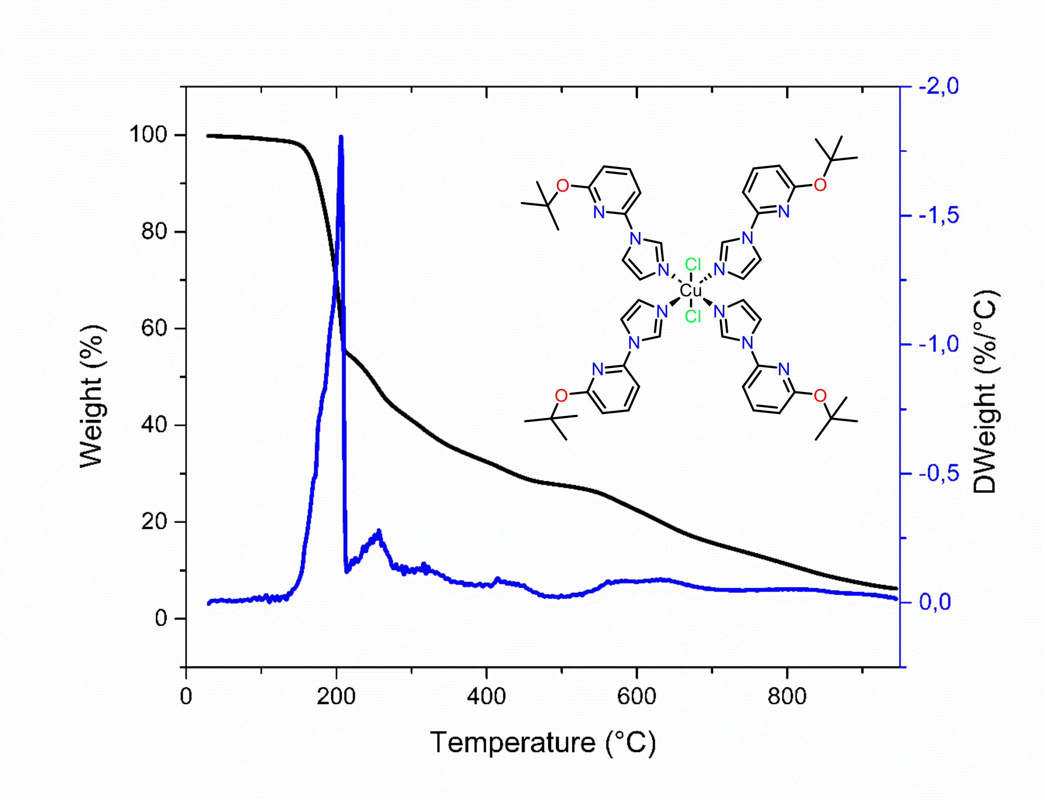

Figure S20. Thermogram of **C2**.

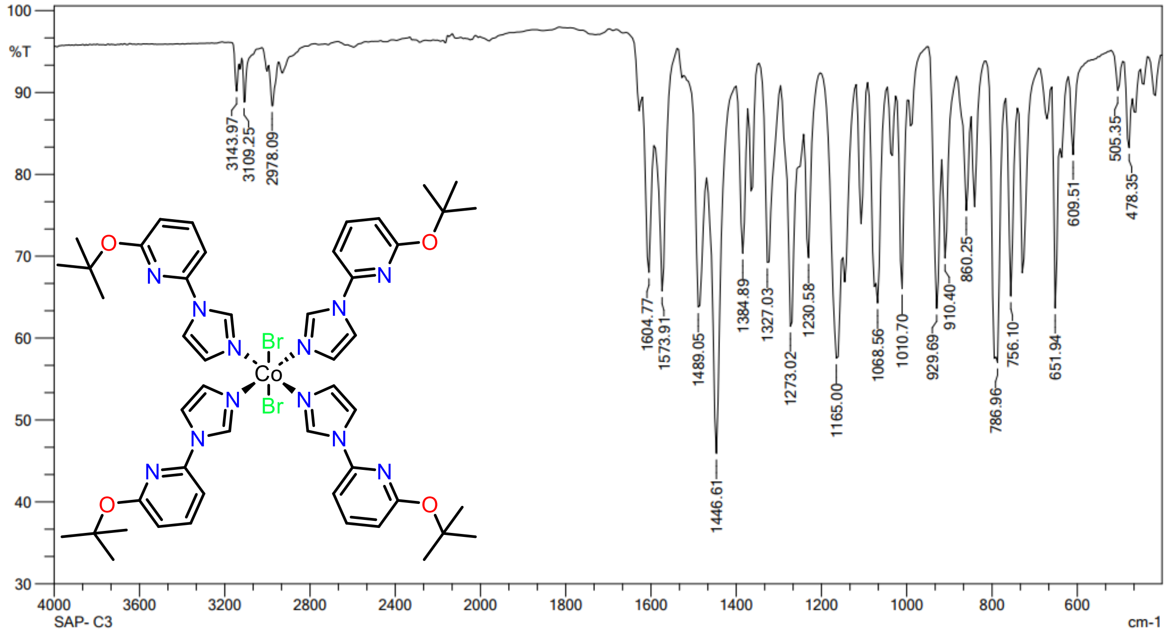

Figure S21. FT-IR spectrum of **C3**.

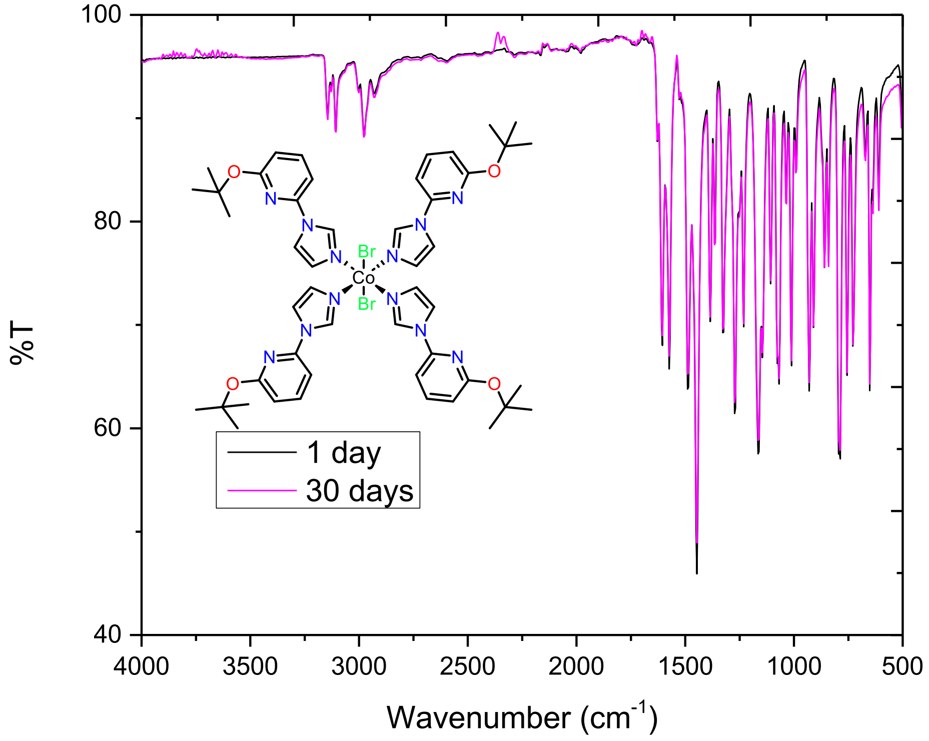

Figure S22. FT-IR spectrum of **C3** for air-stability.

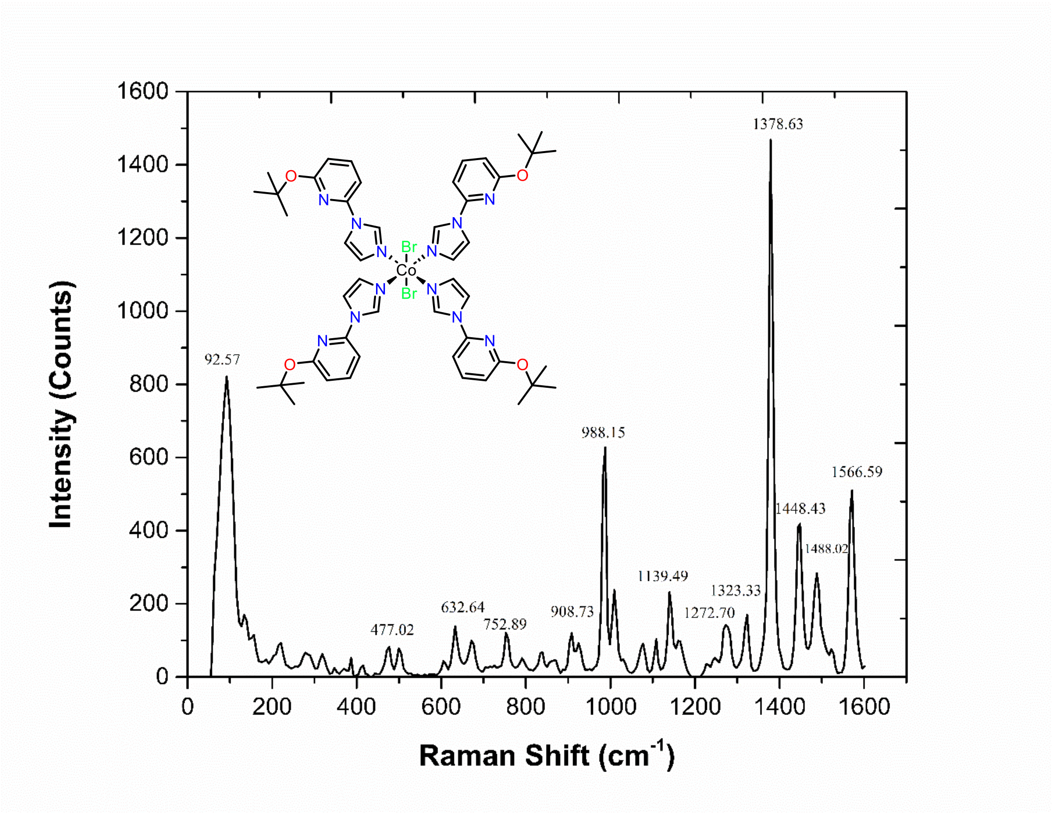

Figure S23. Raman spectrum of **C3**.

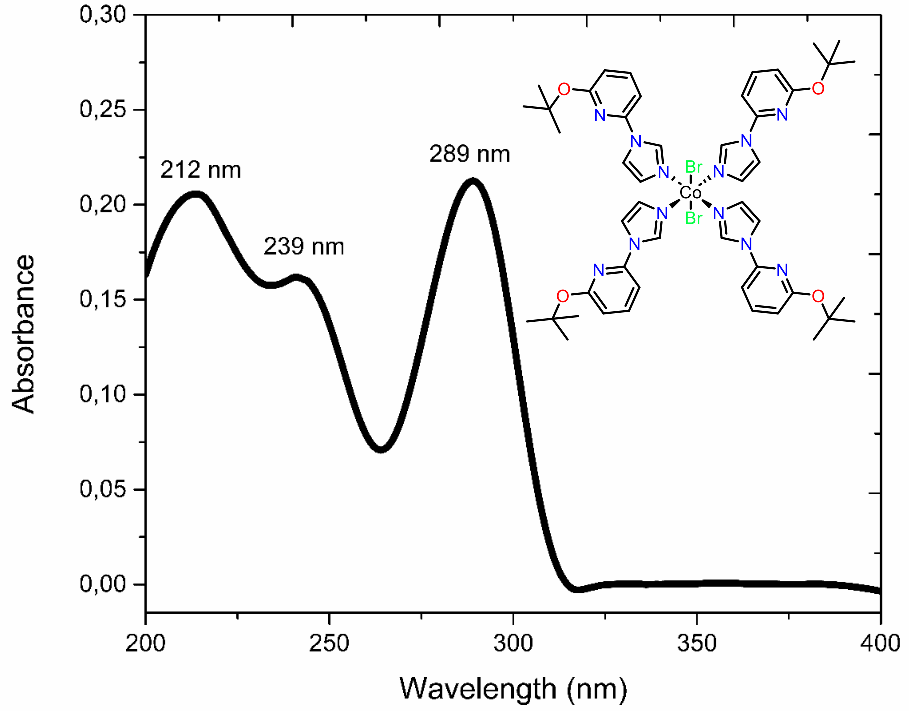

Figure S24. Absorption spectrum of **C3** in DCM (5 x 10^{-6} M).

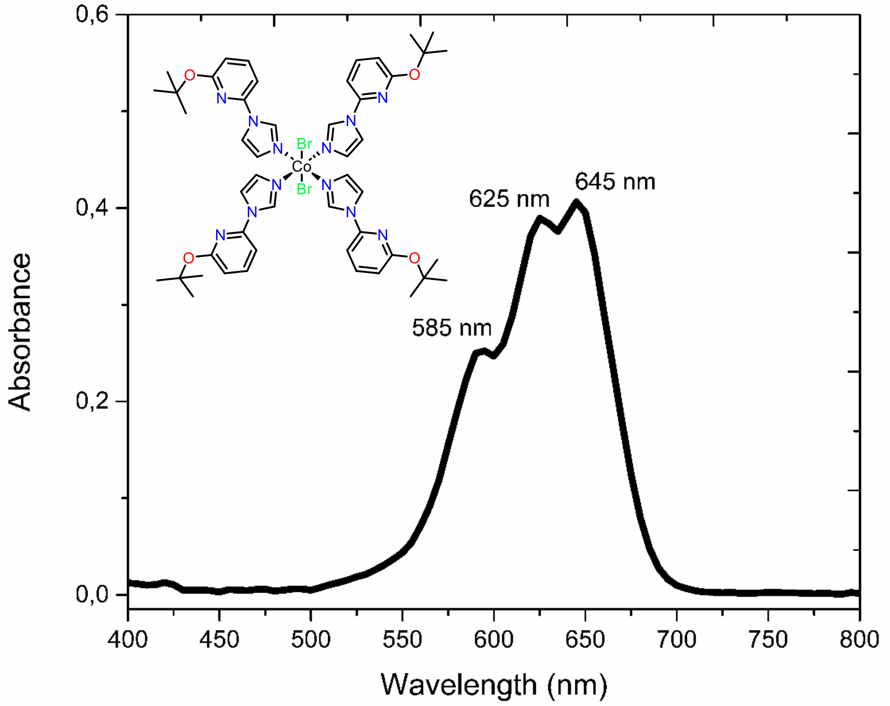

Figure S25. Visible Absorption spectrum of **C3** in DCM (5 x 10^{-4} M).

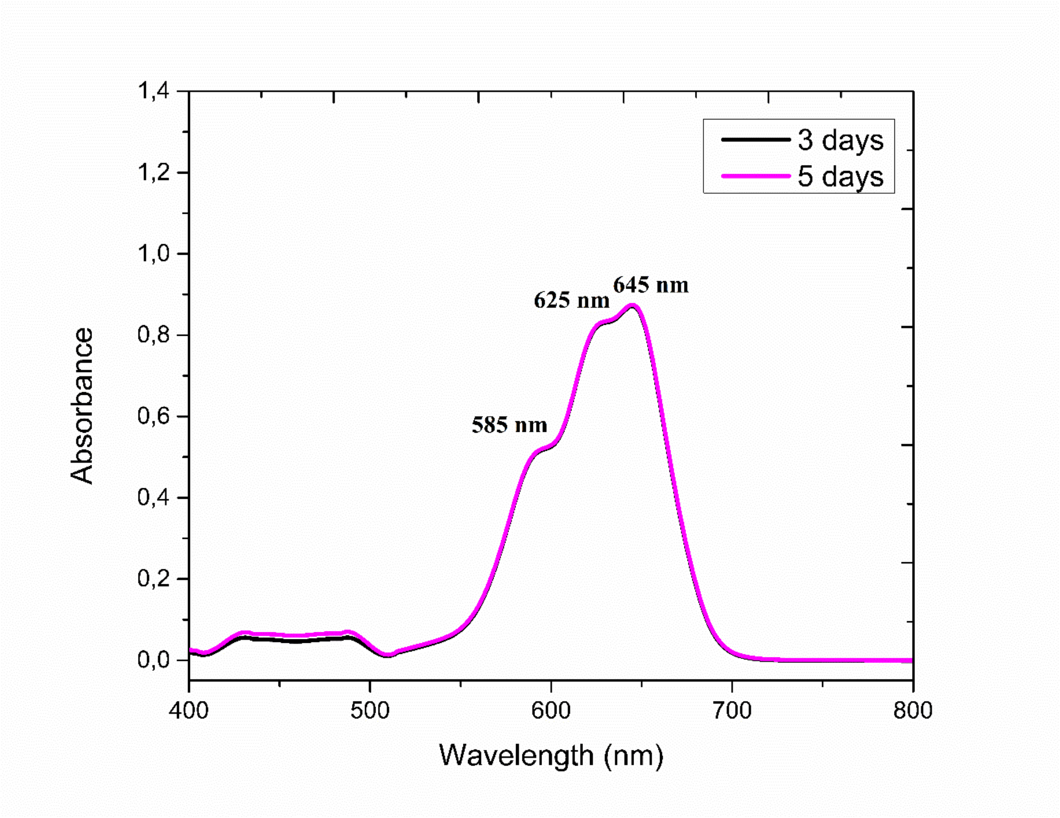

Figure S26. Visible Absorption spectrum of **C3** in DCM (1 x 10^{-3} M) for stability in solution.

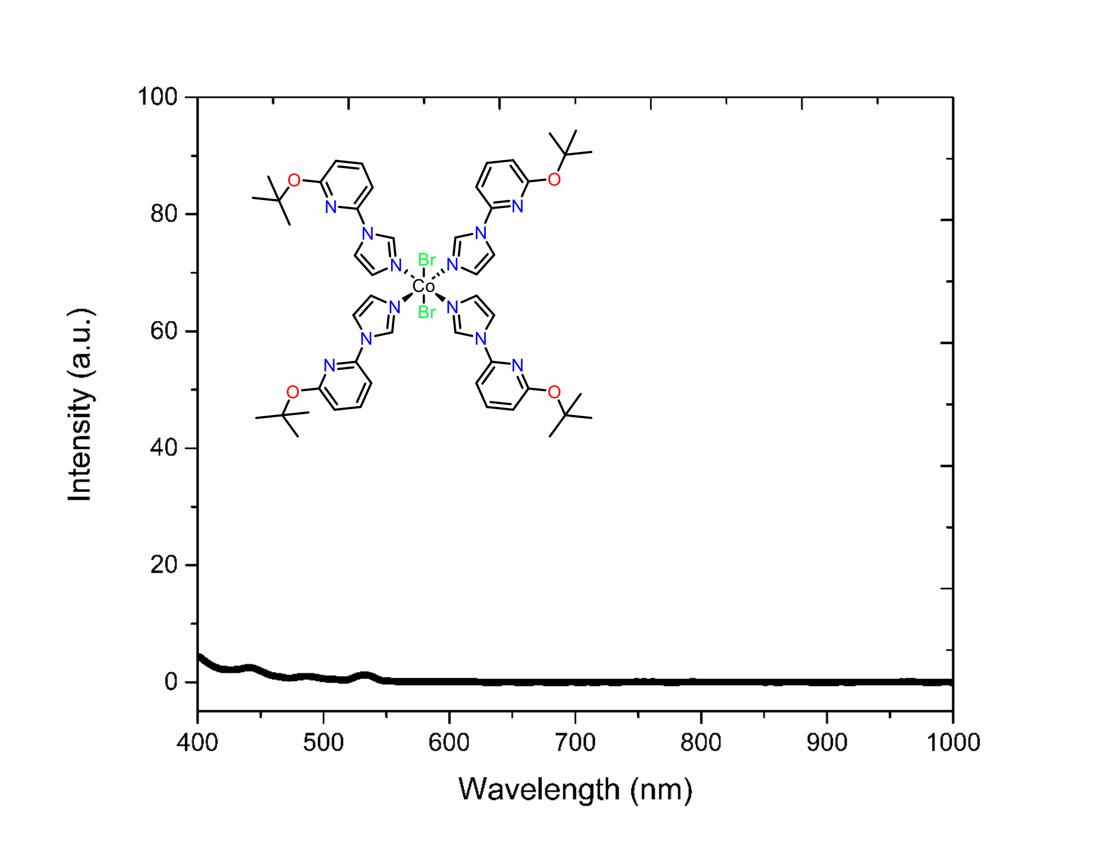

Figure S27. Emission spectrum of **C3** (λ_{em}: 390 nm).

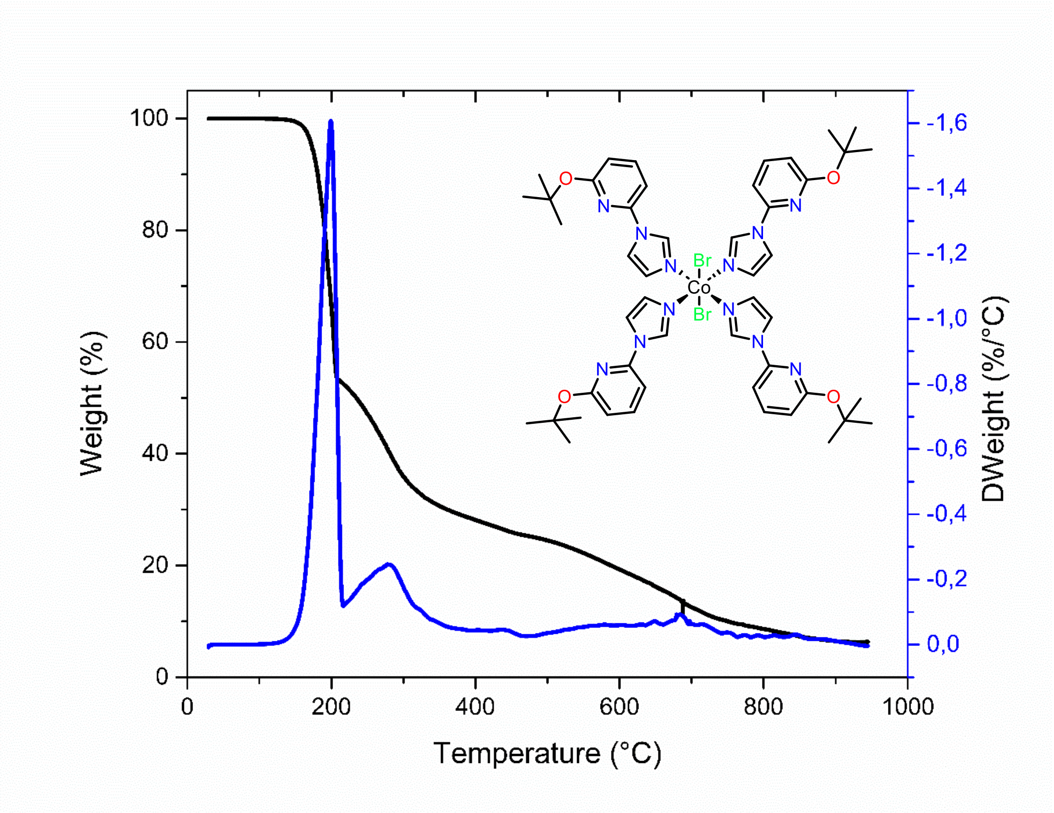

Figure S28. Thermogram of **C3**.

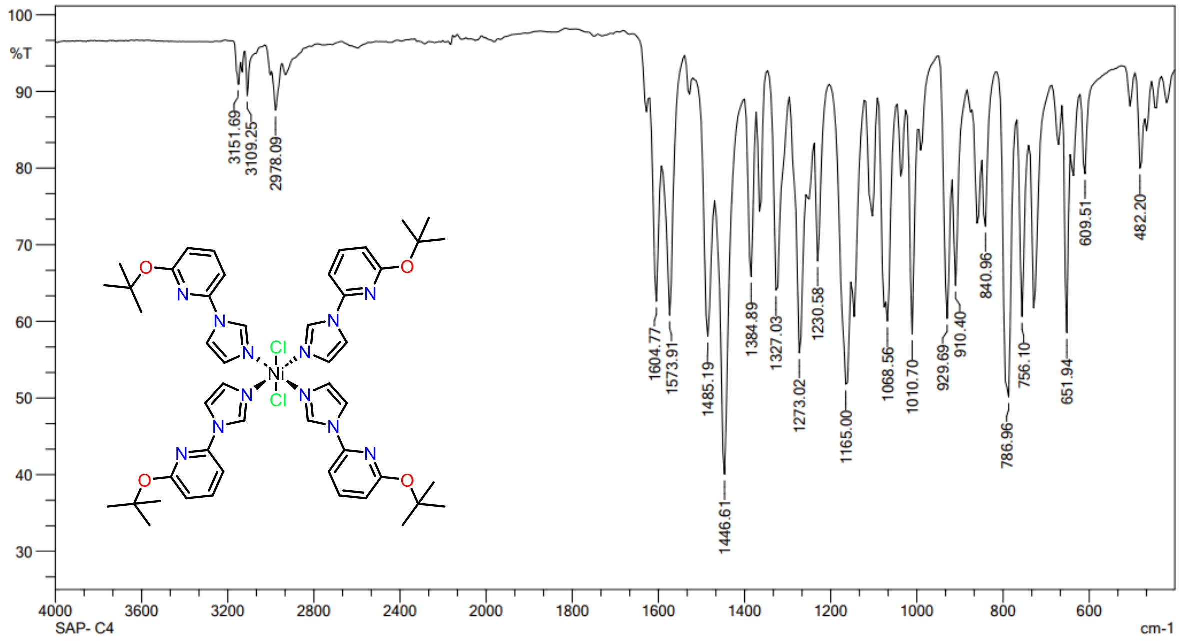

Figure S29. FT-IR spectrum of **C4**.

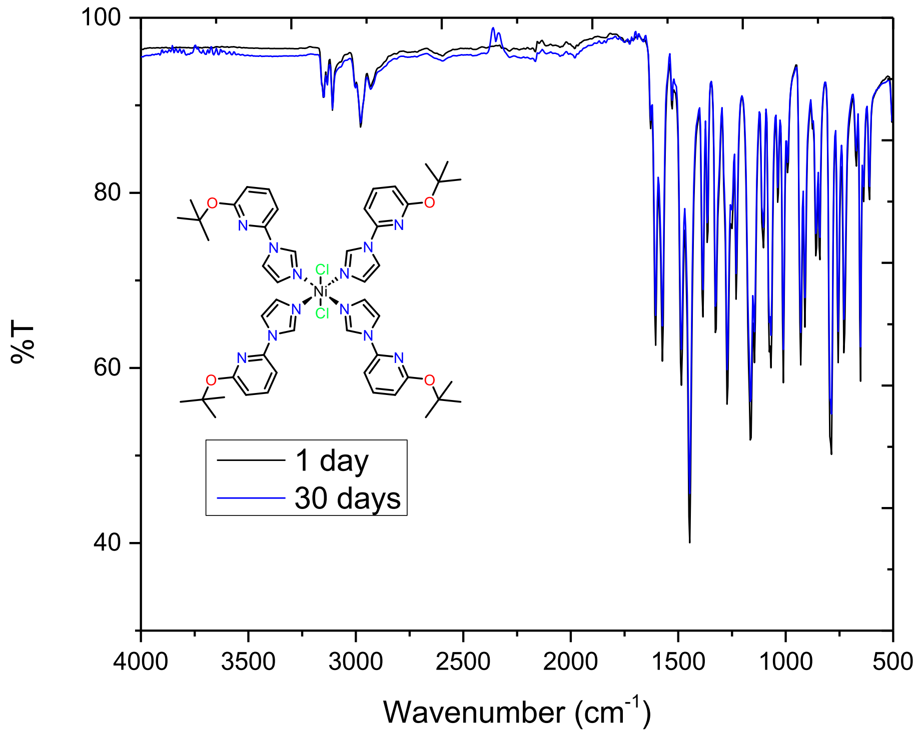

Figure S30. FT-IR spectrum of **C4** for air-stability.

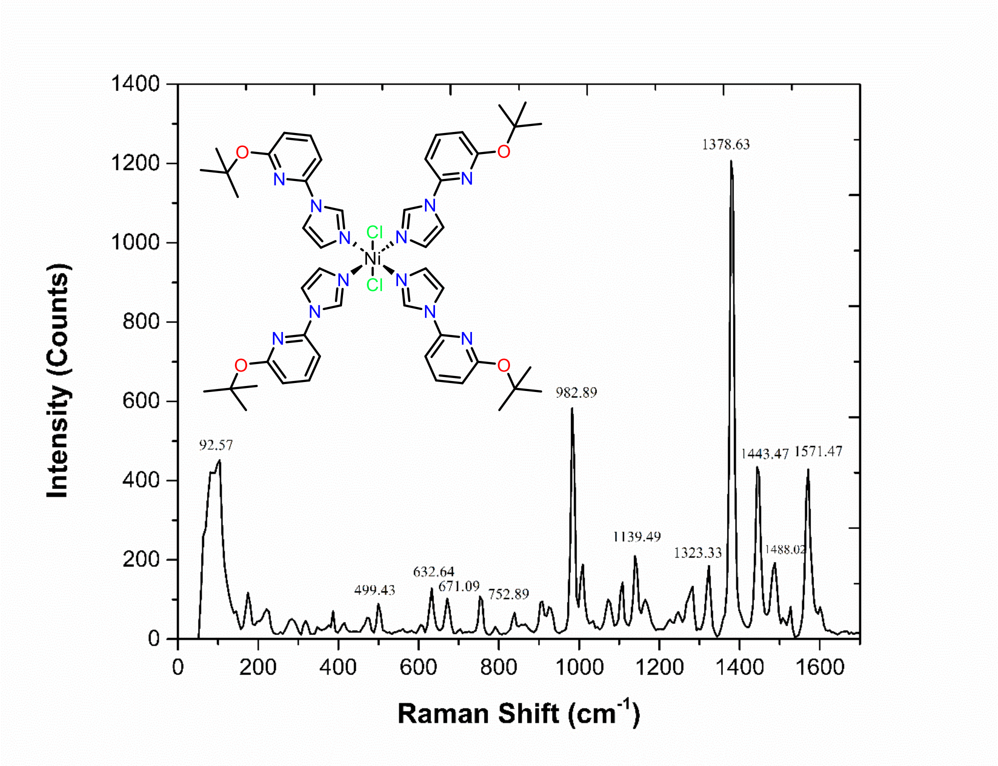

Figure S31. Raman spectrum of **C4**.

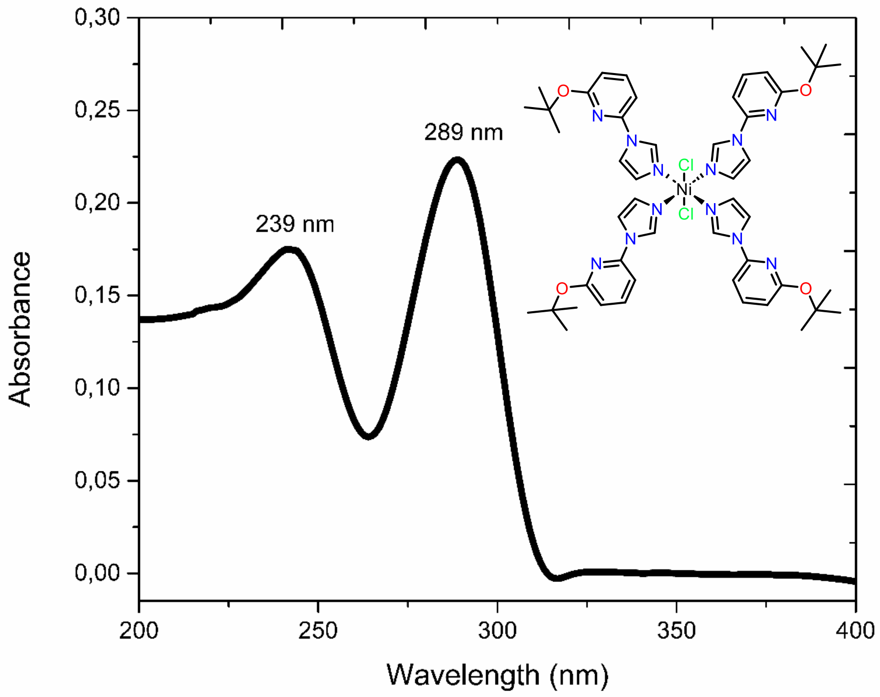

Figure S32. UV Absorption spectrum of **C4** in DCM (5 x 10^{-6} M).

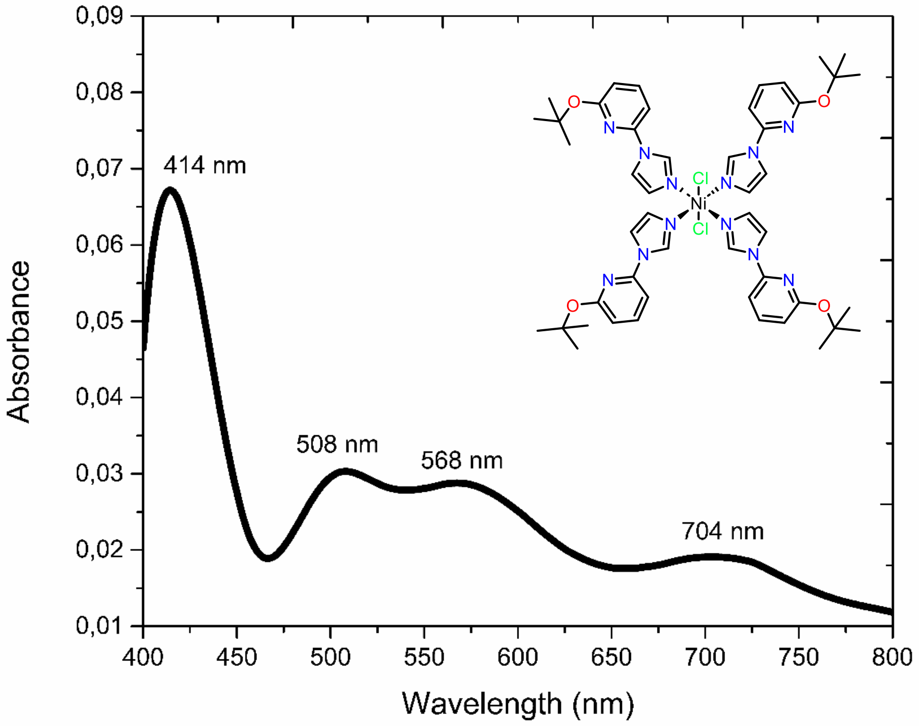

Figure S33. Visible Absorption spectrum of **C4** in DCM (1 x 10^{-3} M).

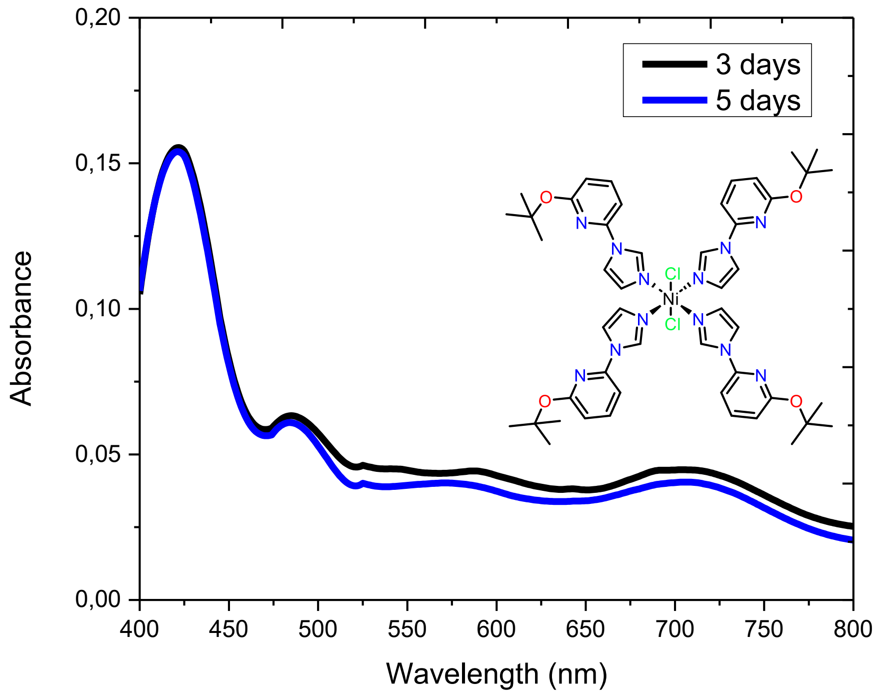

Figure S34. Visible Absorption spectrum of **C4** in DCM (1 x 10^{-3} M) for stability in solution.

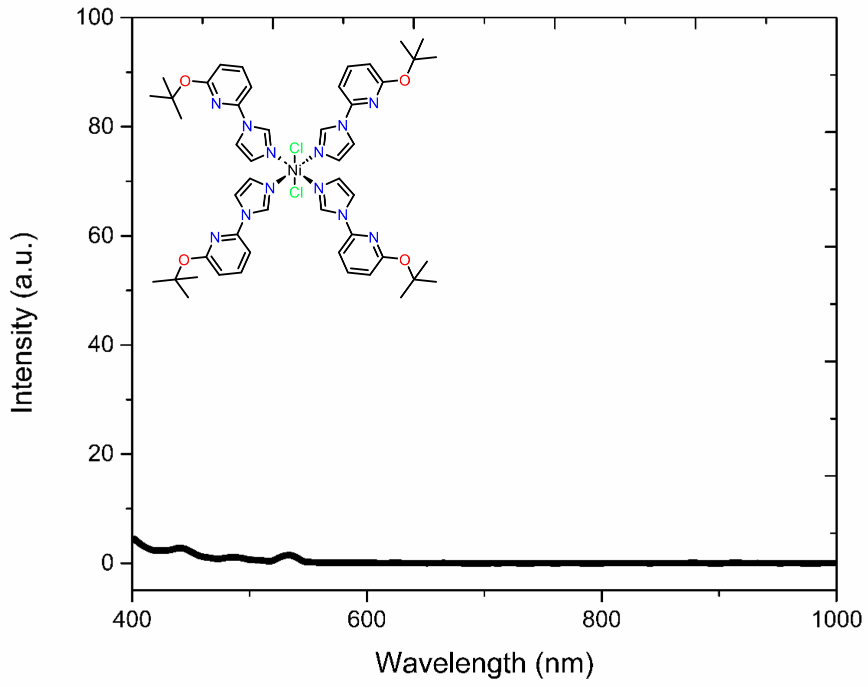

Figure S35. Emission spectrum of **C4** (λ_{em}: 390 nm).

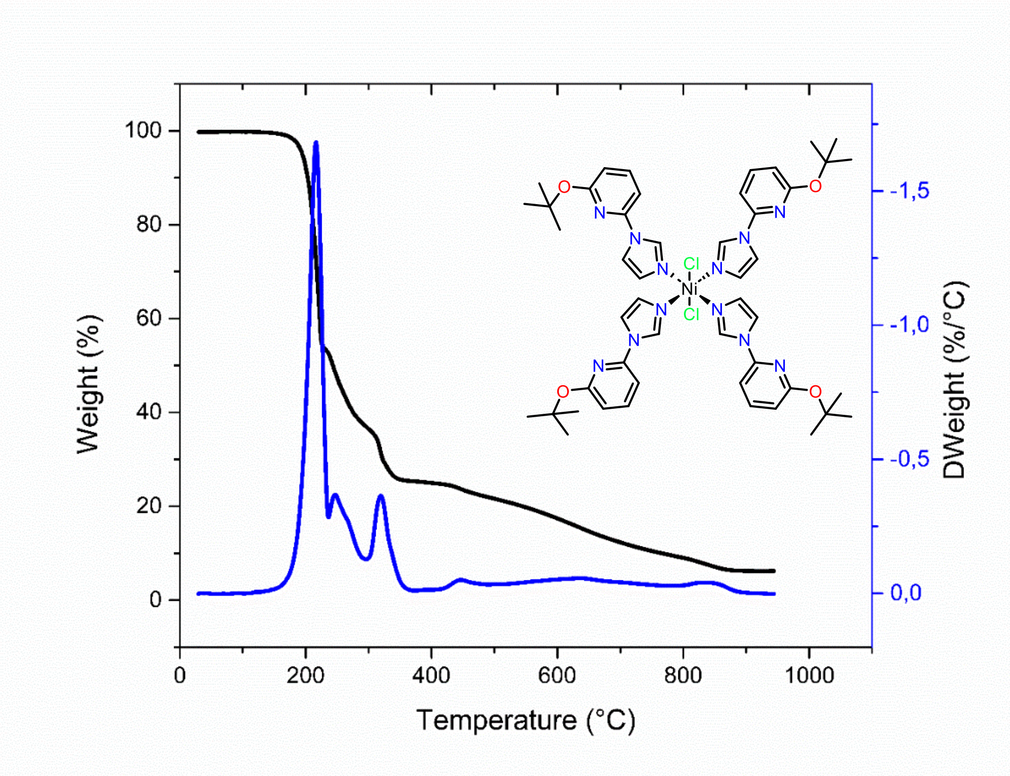

Figure S36. Thermogram of **C4**.

Table S1. Main bands of **L** and **C1-C4** in FTIR and Raman.

Compound	FTIR (cm^{-1})							Raman (cm^{-1})	
	υ(C-H) (aromatic)	υ(-CH_3)	υ(C=C)	υ(C=N)	υ(C-O-C)	υ(C-N)	δ(ring)	M-N	M-X
**Ligand (L)**	3148	2978	1605	1570	1273	1157	1011	-	-
**C1 [$Co(Cl)_2(L)_4$]**	3148	2978	1605	1574	1273	1165	1011	505	471
**C2 [$Cu(Cl)_2(L)_4$]**	3132	2978	1609	1570	1269	1168	1011	402	288
**C3 [$Co(Br)_2(L)_4$]**	3144	2978	1605	1574	1273	1165	1011	477	221
**C4 [$Ni(Cl)_2(L)_4$]**	3152	2978	1605	1574	1273	1165	1011	387	221

Table S2. Main UV-Vis transitions of **C1-C4**

Compound	Band assignation λ (log ε)/nm ($M^{-1}\cdot cm^{-1}$)					
	n-π*(C=N)	π-π*(C=C)	TC-band	λ_2	λ_3	λ_4
**Ligand (L)**	243	289	-	-	-	-
**C1 [$Co(Cl)_2(L)_4$]**	239 (4.533)	289 (4.647)	-	575 (2.736)	610 (2.930)	-
**C2 [$Cu(Cl)_2(L)_4$]**	239 (4.460)	289 (4.541)	-	-	-	730 (2.053)
**C3 [$Co(Br)_2(L)_4$]**	239 (4.512)	289 (4.637)	-	585 (2.651)	625 (2.886)	645 (2.910)
**C4 [$Ni(Cl)_2(L)_4$]**	239 (4.542)	289 (4.660)	414 (1.820)	508 (1.491)	568 (1.447)	704 (1.279)

Table S3. Assignments of the main mass losses of **C1**-**C4**

Compound	Range TGA / °C	Weight lost /% Calcd. (Theoretical)	Assignment
**C1** **[Co(Cl)$_2$(L)$_4$]**	177.06 - 207.22	50.83 (50.60)	2**L** + 2**Cl**
	238.17 - 274.98	15.90	Partial loss of **L**
	582.19 - 619.00	11.92	Partial loss of L
**C2** **[Cu(Cl)$_2$(L)$_4$]**	182.35 - 208.32	45.75 (46.83)	2**L** + **Cl**
	238.93 - 267.00	12.03	Partial loss of **L** + **Cl**
	308.66 - 338.52	7.41	Partial loss of **L**
	410.38 - 451.06	4.87	Partial loss of **L**
	556.67 - 670.95	13.50	Partial loss of **L**
**C3** **[Co(Br)$_2$(L)$_4$]**	183.78 - 205.73	48.53 (47.29)	2**L** + **Br**
	251.47 - 305.39	21.38	Partial loss of **L** + **Br**
**C4** **[Ni(Cl)$_2$(L)$_4$]**	205.99 - 224.46	46.88 (47.06)	2**L** + **Cl**
	238.68 - 262.60	15.38	Partial loss of **L** + **Cl**
	313.13 - 329.59	11.79	Partial loss of **L**

4. Crystallographic data.

Table S4. Crystallographic data of **C1**, **C2**, **C3** and **C4**

**Crystal data**	**C1**	**C2**	**C3**	**C4**
Chemical formula	$C_{48}H_{60}Cl_2CoN_{12}O_4$	$C_{48}H_{60}Cl_2CuN_{12}O_4$	$C_{48}H_{60}Br_2CoN_{12}O_4$	$C_{48}H_{60}Cl_2N_{12}NiO_4$
M_r	998.91	1003.53	1087.81	998.67
Crystal system, space group	Triclinic, *P*-1	Triclinic, *P*-1	Triclinic, *P*-1	Triclinic, *P*-1
Temperature (K)	298(2)	298(2)	298(2)	298(2)
a, *b*, *c* (Å)	9.1131 (8), 11.1406 (12), 14.3218 (9)	9.0464 (11), 11.2375 (14), 14.1769 (15)	9.2552 (6), 11.0876 (8), 14.3421 (6)	9.0552 (8), 11.1394 (10), 14.2940 (8)
α, β, γ (°)	92.869 (7), 102.003 (6), 111.322 (9)	93.671 (9), 101.694 (10), 111.646 (12)	92.809 (5), 101.513 (5), 111.595 (6)	93.337 (6), 101.870 (7), 111.083 (8)
V (Å^3)	1312.2 (2)	1296.4 (3)	1328.75 (15)	1302.69 (19)
Z	1	1	1	1
Radiation type	Cu *K*α	Cu *K*α	Cu *K*α	Cu *K*α
μ (mm^{-1})	3.93	1.97	4.72	1.91
Calculated Density (g cm^{-3})	1.2641(2)	1.2854(3)	1.3595(2)	1.2730(2)
		**Data collection**		
Diffractometer	SuperNova, Dual, Cu at zero, Atlas			

Absorption correction	Multi-scan *CrysAlis PRO* 1.171.41.119a (Rigaku Oxford Diffraction, 2021)			
T_{min}, T_{max}	0.781, 1.000	0.587, 1.000	0.879, 1.000	0.862, 1.000
No. of measured, independent and observed [$I > 2\sigma(I)$] reflections	15067, 5427, 5064	11631, 5334, 4554	22182, 5543, 5355	15482, 5371, 4723
R_{int}	0.038	0.060	0.042	0.040
$(\sin \theta/\lambda)_{max}$ (Å^{-1})	0.630	0.631	0.631	0.631
**Refinement**				
$R[F^2 > 2\sigma(F^2)]$, $wR(F^2)$, S	0.040, 0.109, 1.03	0.064, 0.194, 1.00	0.035, 0.090, 1.07	0.042, 0.123, 1.06
No. of reflections	5427	5334	5543	5371
No. of parameters	311	311	310	310
No. of restraints	6	6	12	6
H-atom treatment	H-atom parameters constrained			
$\Delta\rho_{max}$, $\Delta\rho_{min}$ (e Å^{-3})	0.22, −0.36	0.54, −0.65	0.31, −0.88	0.28, −0.53

5. Biological activity

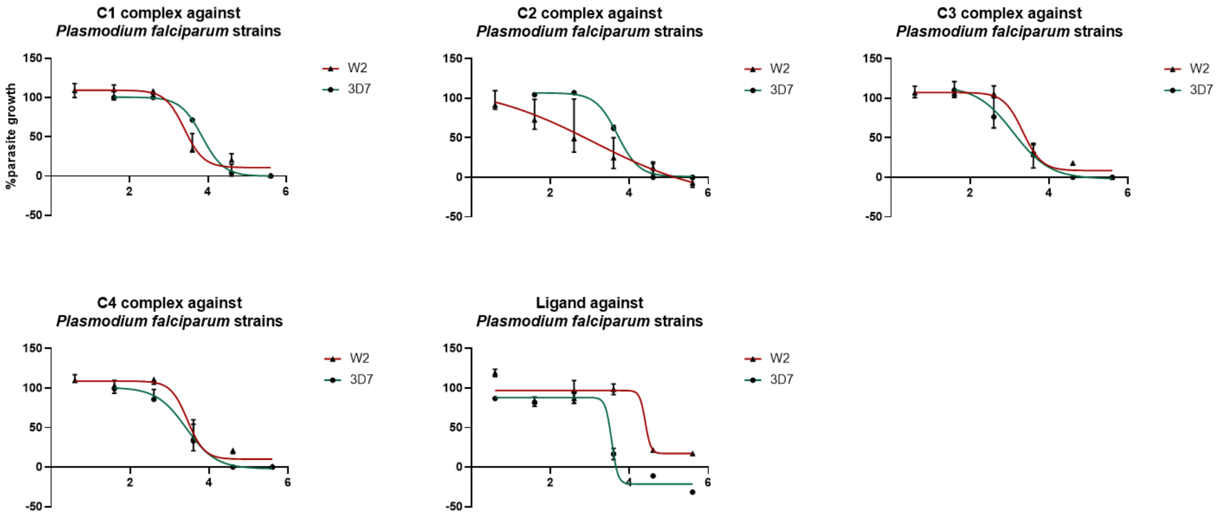

Figure S37. IC_{50} values of the compounds against *Plasmodium falciparum* 3D7 (chloroquine-sensitive) and W2 (chloroquine-resistant). Data are shown as mean ± SD ($\log_{10}$ scale).

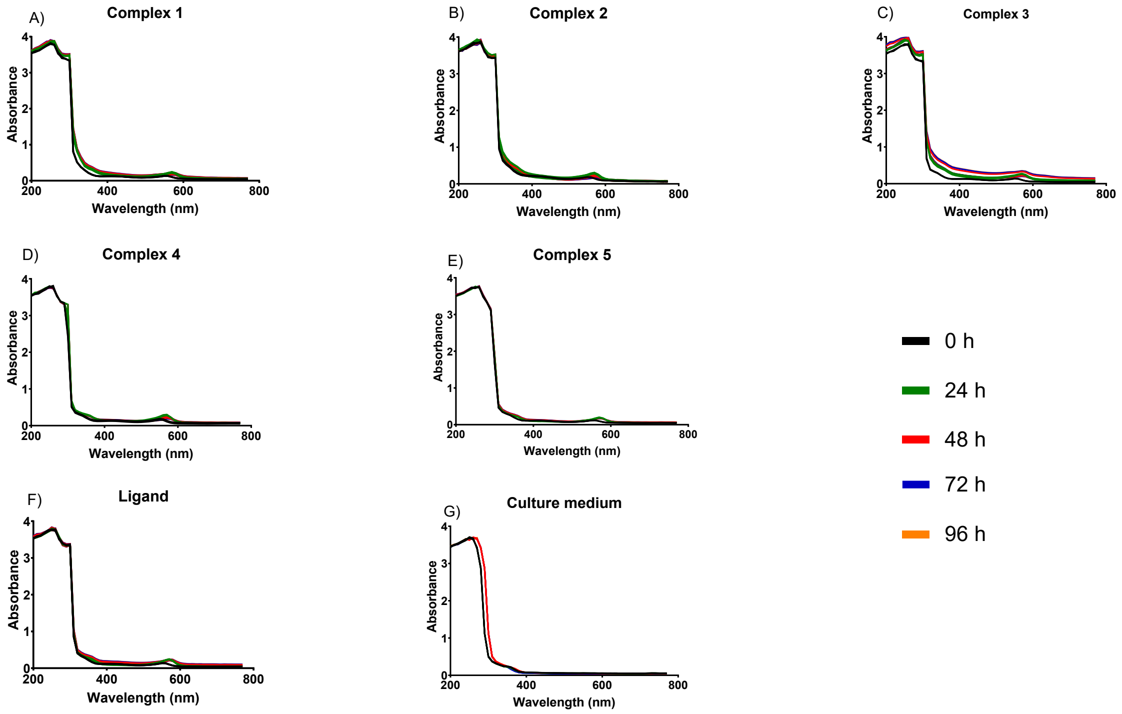

Figure S38. Time-dependent UV–Vis stability assessment of the evaluated compounds and culture medium. Spectra were recorded in the 200–780 nm range at 0, 24, 48, 72, and 96 h for (A) Complex 1, (B) Complex 2, (C) Complex 3, (D) Complex 4, (E) Complex 5, (F) the free ligand, and (G) the culture medium. The overall preservation of the spectral profiles throughout the monitoring period supports the stability of the evaluated solutions under the experimental conditions employed.
